# Arabidopsis EMB2788/Npa1L1 is critical for pre‐rRNA processing, ribosome biogenesis, and embryo development

**DOI:** 10.1111/tpj.71114

**Published:** 2026-09-09

**Authors:** Mei Liu, Shuailei Wang, Zhuoxuan Li, Zhaojiao Liu, Mengjuan Tong, Aiwei Zhang, Nan Chen, Yirui Zhu, Jingsheng Zhang, Lifen Chen, Yi Guo, Rui Li

**Affiliations:** ^1^ Hebei Research Center of the Basic Discipline of Cell Biology Hebei Normal University Shijiazhuang China; ^2^ Ministry of Education Key Laboratory of Molecular and Cellular Biology Hebei Normal University Shijiazhuang China; ^3^ Hebei Collaboration Innovation Center for Cell Signaling and Environmental Adaptation Hebei Normal University Shijiazhuang China; ^4^ Hebei Key Laboratory of Molecular and Cellular Biology Hebei Normal University Shijiazhuang China; ^5^ College of Life Sciences Hebei Normal University Shijiazhuang China

**Keywords:** Npa1L1, pre‐rRNA processing, ribosome biogenesis, embryo development, Arabidopsis

## Abstract

Ribosome assembly, a fundamental process for cellular functions, requires the precise processing of precursor rRNA (pre‐rRNA) into mature rRNAs, a transformation guided by ribosome biogenesis factors (RBFs). While this process is well characterized in yeast and mammals, the roles of RBFs in pre‐rRNA processing in plants remain poorly understood. Here, we report the characterization of Arabidopsis EMB2788, previously linked to embryonic development but functionally uncharacterized. We identified EMB2788 as the homolog of yeast nucleolar pre‐ribosomal‐associated protein 1 (Npa1p), a key pre‐rRNA processing regulator, and named it Npa1L1. *npa1l1* embryos exhibited severe defects from the late globular stage onward, including altered cell division planes, slowed division rates, and a consequent disruption of bilateral symmetry, accompanied by delayed endosperm development, although a small proportion of embryos developed into morphologically abnormal seedlings. Npa1L1 was likely a nucleolar protein and its loss of function led to an increased abundance of 40S subunits and a reduced abundance of 60S subunits and 80S monosomes. Molecular analysis showed pronounced accumulation of 35S pre‐rRNA and 18S rRNA precursors (P‐A_3_) and a reduction in mature 18S rRNA levels. Furthermore, co‐immunoprecipitation experiments indicated that Npa1L1 physically interacted with ribosome biogenesis factors, including the DEAD‐box RNA helicases RH7 and RH27, and the GTPase NSN1. Collectively, our findings uncover that Npa1L1 is a crucial ribosome biogenesis factor in Arabidopsis that contributes to pre‐rRNA processing, ribosomal subunit homeostasis, and embryo development. Nevertheless, the precise function of Npa1L1 in rRNA processing, and whether it differs from that of yeast Npa1p, requires further investigation.

## Significance Statement

Pre‐rRNA processing is crucial for ribosome biogenesis. Arabidopsis Npa1L1, a key ribosome biogenesis factor, functions in pre‐rRNA processing, ribosome assembly, and embryonic patterning.

## INTRODUCTION

Proteins are essential macromolecules that perform diverse and critical functions in living organisms. Ribosomes, the central machinery for protein synthesis, are fundamental to all life processes (Petrov et al., 2014). Defects in ribosome assembly or function are associated with severe human (*Homo sapiens*) disorders known as ribosomopathies (Narla & Ebert, 2010), and in plants, lead to gametophyte abortion, embryonic lethality, or impaired growth and development (Byrne, 2009; Horiguchi et al., 2011; Weis et al., 2015). Eukaryotic ribosomes are large ribonucleoprotein complexes composed of the 40S small ribosomal subunit (SSU) containing the 18S rRNA and the 60S large ribosomal subunit (LSU) which consists of the 25/28S, 5.8S, and 5S rRNAs. Ribosomal RNA serves as both the structural and catalytic core of the ribosome, facilitating peptide bond formation, providing binding sites for tRNAs and translation factors, and engaging with mRNA during protein synthesis (Cech, 2000). Mature rRNAs are derived through extensive splicing and modification of a primary pre‐rRNA transcript, a process that necessitates the assistance of a multitude of ribosome biogenesis factors (RBFs). Thus, understanding the role of RBFs in pre‐rRNA processing is essential for revealing key aspects of ribosome biogenesis and function (Weis et al., 2015).

In eukaryotic cells, ribosome biogenesis begins with the transcription of the 35S/45S rRNA precursor by RNA polymerase I. This polycistronic transcript contains the sequences of the 18S, 5.8S, and 25S rRNAs, flanked and separated by external and internal transcribed spacers (ETS and ITS) (Gruendler et al., 1989; Saez‐Vasquez & Delseny, 2019; Unfried et al., 1989; Unfried & Gruendler, 1990). In contrast, the 5S rRNA is independently transcribed by RNA polymerase III (Layat et al., 2012). The maturation of pre‐rRNA involves the coordinated removal of these spacer sequences through a series of endo‐ and exonucleolytic cleavage steps, facilitated by a large set of ribosome biogenesis factors (RBFs) (Henras et al., 2015; Tomecki et al., 2017; Weis et al., 2015). Yeast studies have revealed a pre‐rRNA processing pathway where the 35S pre‐rRNA undergoes initial cleavage in the 5'ETS, followed by secondary cleavage in ITS1 to yield 18S rRNA precursors and 27S rRNA precursors (Klinge & Woolford, 2019; Tomecki et al., 2017; Woolford Jr. & Baserga, 2013). Human studies have uncovered two pre‐rRNA processing pathways: In the major pathway, initial cleavage occurs at the ITS1 of 45S pre‐rRNA to produce 18S rRNA precursors and 27S rRNA precursors, after which the 5'ETS is trimmed from the 18S rRNA precursors. In the minor pathway, cleavage first takes place within the 5'ETS, and a subsequent cleavage event at ITS1 generates 18S rRNA precursors and 27S rRNA precursors (Aubert et al., 2018; Tomecki et al., 2017). However, compared to the well‐characterized systems in yeast and humans, the understanding of pre‐rRNA processing in plants remains less advanced (Weis et al., 2015). Plant pre‐rRNA processing exhibits several distinctive features; for instance, the 5'ETS in Arabidopsis is substantially longer than its yeast counterpart and contains additional processing sites (Caparros‐Ruiz et al., 1997; Sáez‐Vasquez et al., 2004). Arabidopsis employs three distinct pre‐rRNA processing pathways: a minor 5'ETS‐first pathway (similar to yeast), a major ITS1‐first pathway (similar to the major pathway in humans), and a plant‐specific ITS2‐first pathway (Hang et al., 2014; Saez‐Vasquez & Delseny, 2019; Weis et al., 2015; Zakrzewska‐Placzek et al., 2010). The ITS2‐first pathway involves initial cleavage at the C_2_ site within ITS2; however, its molecular mechanisms and regulation remain poorly understood (Palm et al., 2019).

Furthermore, although Arabidopsis possesses numerous RBFs homologous to those in yeast, their functions often diverge, and plants also encode lineage‐specific RBFs (Palm et al., 2019; Weis et al., 2015). To date, only a limited number of plant RBFs have been functionally characterized. For example, Arabidopsis Protein Arginine Methyltransferase 3 (AtPRMT3) promotes the major ITS1‐first pathway while repressing the minor 5'ETS‐first pathway (Hang et al., 2014). AtPRMT3 also acts synergistically with RPS2B to regulate the dynamic assembly and disassembly of the 90S/SSU processome during ribosome biogenesis and helps suppress nucleolar stress (Hang et al., 2021). Another key factor, Nin1 Binding Protein 1 (AtNOB1), functions as an endonuclease required for the processing of P‐A_3_ pre‐rRNA and the maturation of the 40S small ribosomal subunit (Missbach et al., 2013). In rice (*Oryza sativa*), the GTPase Nug2 is essential for the processing of 27S rRNA precursors and biogenesis of the 60S large ribosomal subunit (Im et al., 2011). Similarly, maize (*Zea mays*) ZmURB2 plays a critical role in the cleavage of 35S, P‐A_3_, and 18S‐A_3_ pre‐rRNAs and is necessary for the production of both ribosomal subunits (Wang et al., 2018). Thus, more crucial RBFs involved in plant rRNA processing remain to be explored.

To identify key genes involved in plant sexual reproduction, we previously screened Arabidopsis mutants exhibiting abnormal segregation ratios in self‐pollination that significantly deviated from the expected 3:1 ratio. Among these, we isolated a loss‐of‐function mutant of EMB2788, which had previously been implicated in embryonic development (Tzafrir et al., 2004), though its biochemical function and mechanistic role remained unknown. Bioinformatic analysis revealed that the EMB2788 protein, with a coding sequence of 10 168 bp, encodes a 2574‐amino acid polypeptide containing an Npa1 (nuclear pre‐ribosomal‐associated protein 1) domain and a functionally uncharacterized NopRA1 domain (source: InterPro). In yeast, nucleolar pre‐ribosomal associated protein 1 (Npa1p) is a core factor essential for ribosome biogenesis and plays a critical role in the processing of 27SA_2_ pre‐rRNA (Dez et al., 2004; Joret et al., 2018; Rosado & De La Cruz, 2004). Npa1p forms a complex with Npa2p, Dbp6p, Nop8p, and Rsa3p, which associates with pre‐60S particles and is required for the assembly of this complex (Joret et al., 2018; Rosado et al., 2007). In human cells, yeast Npa1p's homolog URB1 ensures proper 3′ external transcribed spacer removal from pre‐rRNA, preventing exosome‐mediated degradation and ensuring 28S rRNA maturation and 60S subunit biogenesis (Shan et al., 2023). Notably, loss of function of unhealthy ribosome biogenesis 1 (Urb1), the zebrafish (*Danio rerio*) homolog of Npa1p, results in reduced levels of 40S and 60S subunits, as well as 80S monosomes, and leads to developmental abnormalities (He et al., 2017).

In this study, we found that the previously uncharacterized Arabidopsis protein EMB2788, hereafter designated Npa1L1, functions as a ribosome biogenesis factor (RBF) involved in rRNA processing. Mutation of *Npa1L1* resulted in increased abundance of 40S subunits and a reduced abundance of 60S subunits and 80S monosomes. Meanwhile, 35S pre‐rRNA and 18S rRNA precursors accumulated, and mature 18S rRNA production was reduced. We further showed that Npa1L1 likely formed a complex with DEAD‐box RNA Helicase 7 (RH7), RNA Helicase 27 (RH27), and Nucleostemin‐like 1 (NSN1). Morphological analysis of *npa1l1* mutants revealed aberrant asymmetric cell division in globular stage embryos, leading to cotyledon asymmetry and delayed development.

## RESULTS

### EMB2788 protein, which has an Npa1 domain implicated in ribosomal RNA processing, is redesignated as Npa1‐like 1

To identify genes essential for sexual reproduction in Arabidopsis, we performed a forward genetic screen by transforming wild‐type (WT) plants with a *pLAT52*::*GUS* reporter. We identified a line exhibiting a self‐pollination segregation ratio significantly deviating from the expected 3:1 Mendelian ratio. Thermal asymmetric interlaced PCR revealed that this phenotype was caused by a mutation in the *EMBRYO DEFECTIVE 2788* (*EMB2788*) gene. Although initially characterized by the Seed Genes Project as essential for embryonic development (Tzafrir et al., 2004), the molecular function and mechanistic role of EMB2788 remained unknown.

Bioinformatic analysis using InterPro predicted that the large 289 kDa EMB2788 protein contained an N‐terminal Npa1 (nuclear pre‐ribosomal‐associated protein 1) domain and a C‐terminal NopRA1 domain, both implicated in ribosome biogenesis (Figure S1a). Protein sequence alignment revealed that EMB2788 shared homology with an Arabidopsis paralog and showed high conservation with *Saccharomyces cerevisiae* Npa1p, a protein involved in ribosomal RNA processing (Figure S2). Yeast Npa1p (203 kDa) contains Npa1 and NopRA1 domains (Figure S1a) (Joret et al., 2018).

Based on this structural and functional homology, we redesignated EMB2788 as Npa1‐like 1 (Npa1L1) and its Arabidopsis homolog as Npa1‐like 2 (Npa1L2), which share an amino acid sequence identity of 66.01% (Figure S3a). The Npa1 and NopRA1 domains of the two paralogs exhibit 69.93% and 71.23% amino acid sequence identity, respectively, indicative of potential functional redundancy (Figure S3a). However, the amino acid identity of the Npa1 domain between Arabidopsis Npa1L1 and yeast Npa1p is 17.72%, while that of the NopRA1 domain is only 15.88% (Figure S3b,c). Expression profiling via the Electronic Fluorescent Pictograph (eFP) browser showed that *Npa1L1* was widely expressed, with elevated transcript levels in shoot apices, embryos, and 24‐hour imbibed seeds (Figure S4a) (Winter et al., 2007). Similarly, *Npa1L2* exhibited a wide expression pattern, with increased expression in shoot apices, embryos, flowers, and mature pollen. Notably, the transcript level of *Npa1L2* was extremely low, approximately one‐thirtieth of that of *Npa1L1* (Figure S4b).

Phylogenetic analysis indicated that Npa1L1 was highly conserved across diverse species, including humans, zebrafish, and *Saccharomyces cerevisiae* (Figure S2). These species all contain Npa1 and NopRA1 domains (Figure S1a). Although overall amino acid identity of the Npa1 and NopRA1 domains was relatively low, key residues such as phenylalanine, leucine, and arginine were strictly conserved (Figure S1b,c).

**Figure 1:**
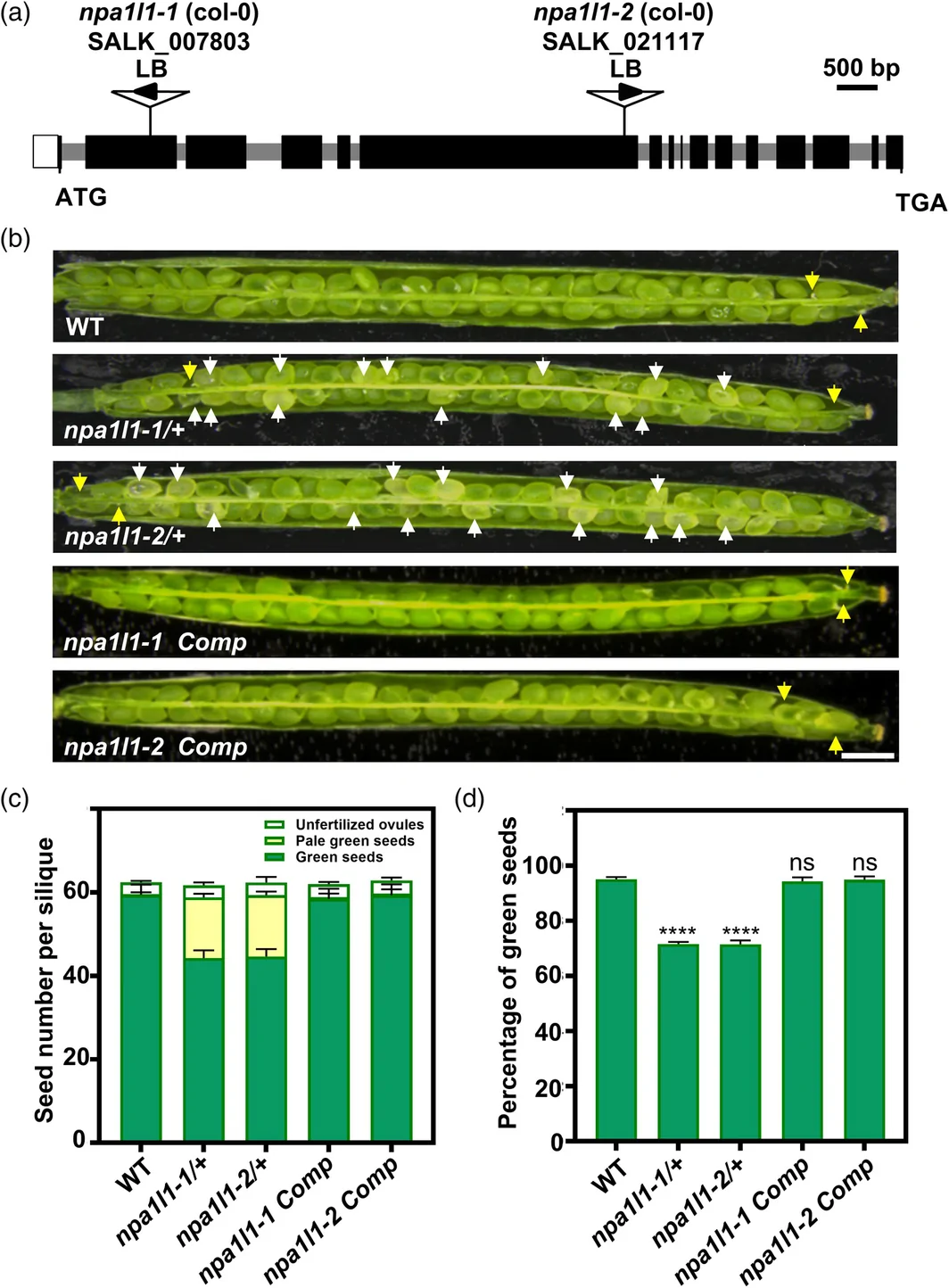
Characterization of the *npa1l1/+* mutants and complementation lines. (a) Schematic diagram of the Npa1L1 genomic sequence structure and T‐DNA insertion sites. Black boxes represent exons, gray lines represent introns, and the white box represents the untranslated region (UTR). (b) Seed development in WT, *npa1l1‐1/+*, *npa1l1‐2/+*, and complemented transgenic plants expressing pNpa1L1::GFP‐Npa1L1 (Comp) in the *npa1l1‐1* and *npa1l1‐2* backgrounds. White arrows indicate pale green seeds and yellow arrows indicate unfertilized ovules. Scale bar = 1 mm. (c) Quantification of seed number per silique (left) and (d) green seeds percentage (right) in the indicated genotypes. Seeds were categorized as green seeds, pale green seeds, or unfertilized ovules. Data are presented as means ± SD (*n* = 25 siliques per genotype). Asterisks indicate a significant difference compared with the WT (Student's *t*‐test, ****P < 0.0001). ns: no significant difference (Student's *t*‐test, *P* > 0.05).

To investigate the biological function of Npa1L1, we characterized two T‐DNA insertion mutants, *npa1l1‐1* (SALK_007803) and *npa1l1‐2* (SALK_021117). The Npa1L1 genomic sequence spans 10 168 bp and comprises 16 exons and 15 introns. The T‐DNA insertions were located in exons 2 and 6, respectively (Figure 1a; Figure S5a). Genotyping was performed on mature seedlings derived from self‐pollinated *npa1l1/+* heterozygous mutants, and no viable homozygous *npa1l1* plants were obtained, suggesting embryonic lethality (Figure S5b). Segregation ratios of self‐pollinated *npa1l1–1/+* and *npa1l1–2/+* progeny were 123:250:0 and 131:258:0 (WT:heterozygote:homozygote), approximating a 1:2:0 ratio (Table 1), which significantly deviated from Mendelian expectation. These results suggest that gametophytic function may be normal, but homozygous mutation of *npa1l1* may disrupt embryonic development.

**Table 1:** Segregation of selfed progeny of the *npa1l1‐1/+* and *npa1l1‐2/+*

Parental genotypes	Progeny genotypes				Expected
Female × Male	*+/+* (1)	*+/–* (2)	*–/–* (1)	*χ* ^2^	*χ* ^2^
*npa1l1‐1/+*⊗	123 (1)	250 (2.03)	0	124.36↑	5.99
*npa1l1‐2/+*⊗	131 (1)	258 (1.97)	0	150.9↑	5.99

*Notes:* Segregation of selfed progeny was performed between *npa1l1‐1/+* and *npa1l1‐2/+* plants. *χ* ^2^ was chi‐square test (*df* = 2, *P* < 0.05, *χ* ^2^ > 5.99). ↑ represents that the actual *χ* ^2^ is increased compared with expected *χ* ^2^.

To investigate the effect of *Npa1L1* dysfunction on seed development, we examined siliques of WT and *npa1l1*/+ plants. WT siliques harbored normal green seeds and unfertilized ovules (yellow arrows). Compared with the WT, heterozygous siliques of *npa1l1‐1/+* and *npa1l1‐2/+* additionally produced many abnormal pale green seeds (white arrows) (Figure 1b). Quantitative analysis revealed that seed number per silique (green + pale green + unfertilized) was comparable across all genotypes (Figure 1c). However, the number of pale green seeds was drastically increased in heterozygous mutants (Figure 1c). Heterozygous mutants of *npa1l1‐1/+* and *npa1l1‐2/+* exhibited approximately 75% green seeds, accounting for nearly 25% abnormal seeds, which was consistent with the expected proportion of homozygous mutants (Figure 1d).

To confirm that the observed abnormal seeds were caused by loss of *Npa1L1* function, we performed a genetic complementation assay. A construct consisting of the native *Npa1L1* promoter (2001 bp upstream of the ATG) driving a GFP‐tagged genomic *Npa1L1* (10 165 bp) was introduced into *npa1l1–1*/+ and *npa1l1–2*/+ plants. The resulting transgenic lines successfully rescued the mutant phenotype: homozygous plants were recovered, and both silique morphology and abnormal seed percentage were restored to WT levels (Figure 1b–d; Figure S5a,d).

### *npa1l1* embryos exhibit improper cell division orientation from the late globular stage, resulting in delayed development and impaired bilateral symmetry

**Figure 2:**
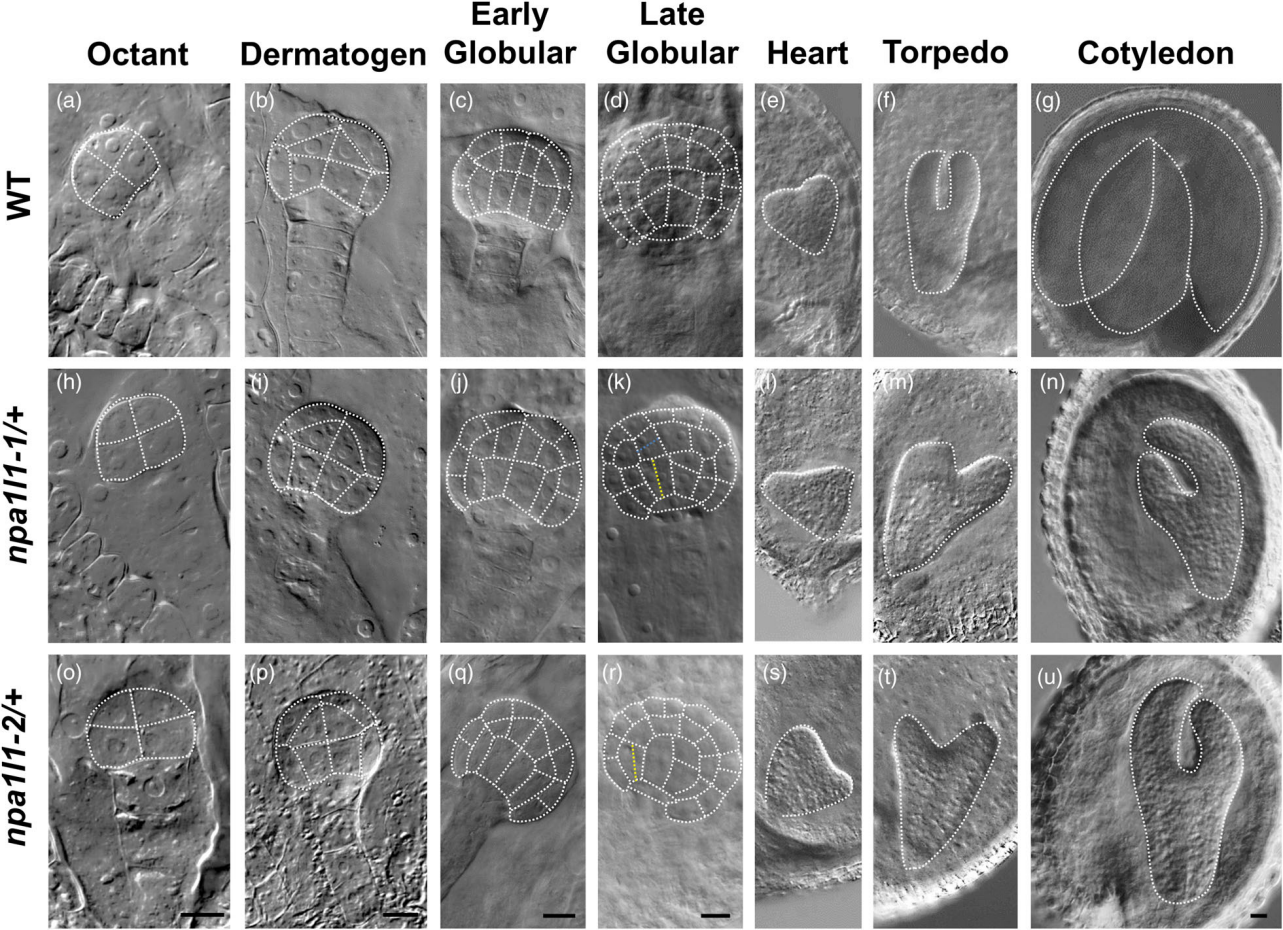
Cell division and bilateral symmetry are impaired in *npa1l1/+* embryos. (a, h, o) Embryos of WT, *npa1l1‐1/+*, and *npa1l1‐2/+* at the octant stage. Scale bar = 10 μm. (b, i, p) Embryos of WT, *npa1l1‐1/+*, and *npa1l1‐2/+* at the dermatogen stage. Scale bar = 10 μm. (c, j, q) Embryos of WT, *npa1l1‐1/+*, and *npa1l1‐2/+* at the early globular stage. Scale bar = 10 μm. (d, k, r) Late globular stage embryos in WT siliques, and late globular stage embryos with abnormal division in *npa1l1‐1/+* and *npa1l1‐2/+* siliques, respectively. Yellow lines indicate the abnormal longitudinal division planes in the inner cells of *npa1l1‐1/+* and *npa1l1‐2/+* embryos. Blue lines indicate the abnormal transverse division planes in the inner cells of *npa1l1‐1/+* embryos. Scale bar = 10 μm. (e, l, s) Heart stage embryos in WT siliques and heart stage embryos with asymmetric cotyledon primordia in *npa1l1‐1/+* and *npa1l1‐2/+* siliques, respectively. (f, m, t) Torpedo stage embryos in WT siliques, and torpedo stage embryos with asymmetrically developing cotyledons in *npa1l1‐1/+* and *npa1l1‐2/+* siliques, respectively. (g) Cotyledon stage embryos in WT siliques. (n, u) Cotyledon stage embryos with asymmetric cotyledons and delayed development in *npa1l1‐1/+* and *npa1l1‐2/+* siliques, respectively. White lines indicate the outline of the embryo proper and the cell division planes. Scale bars for (e–g), (l–n), and (s–u) are identical. Scale bar = 20 μm.

To investigate potential defects in the embryonic development of *npa1l1* homozygotes, the embryo development within siliques of WT, *npa1l1‐1/+*, and *npa1l1‐2/+* plants was examined using whole‐mount clearing followed by differential interference contrast (DIC) microscopy. In the WT, at the zygote stage, an elongated zygote containing a distinct nucleus was observed aligned along the future apical–basal axis (Figure S6a). The first asymmetric division then occurred, resulting in the formation of a smaller apical cell and a larger basal cell (Figure S6b). By the 2/4‐cell stage, the apical cell underwent two longitudinal divisions perpendicular to each other, generating four equal‐sized cells, while the basal cell divided transversely to produce the suspensor, which connected the embryo to maternal tissues (Figure S6c). At the octant stage, a transverse division separated the embryo into upper and lower tiers, forming an eight‐celled structure (Figure 2a). During the dermatogen stage, each cell underwent a tangential division, segregating the embryo proper into four inner and four outer cells (Figure 2b). By the early globular stage, the inner cells divided longitudinally and the outer cells anticlinally, contributing to embryonic patterning (Figure 2c). In both *npa1l1–1/+* and *npa1l1–2/+* mutants, no conspicuous morphological differences were observed compared to the WT across developmental stages from zygote to early globular (Figure S6d–i; Figure 2h–j, o–q).

In WT embryos at the late globular stage, the four inner cells of the lower tier divided transversely, while the outer cells underwent further anticlinal divisions to extend the epidermal layer (Figure 2d). In contrast, embryos of *npa1l1–1/+* exhibited aberrant division patterns: three inner cells of the lower tier divided transversely, while one underwent longitudinal rather than transverse division. Additionally, an ectopic transverse division occurred in one inner cell of the upper tier (Figure 2k). In *npa1l1–2/+* embryos, two lower tier inner cells divided transversely, one divided longitudinally instead of transversely, and one failed to undergo division altogether (Figure 2r).

Given that the lower tier inner cells are precursors to the ground and vascular tissues (Lau et al., 2012; ten Hove et al., 2015), these aberrant divisions suggest impaired formation of these tissues in *npa1l1/+* mutants. These results indicate that divergence in division orientation and a reduced division rate occur in the progenitors of ground and vascular tissues beginning at the late globular stage in *npa1l1* mutants.

Furthermore, subsequent developmental stages were also affected. While WT embryos exhibited symmetrical cotyledon formation at the heart, torpedo, and cotyledon stages (Figure 2e–g), *npa1l1/+* mutants displayed obviously shortened and asymmetrical embryos at the heart, torpedo, and cotyledon stages, indicating disruption of bilateral symmetry (Figure 2l–n, s–u). Collectively, these observations demonstrate that loss of Npa1L1 function perturbs cell division orientation and retards the rate of cytokinesis, ultimately leading to a failure in the establishment of bilateral symmetry during late embryogenesis.

Subsequently, we performed a quantitative analysis of embryonic morphology using siliques collected at 7 days after pollination (DAP) from WT, *npa1l1–1/+*, and *npa1l1–2/+* plants. By this stage, WT embryos had developed nearly synchronously to the cotyledon stage. In contrast, siliques from *npa1l1–1/+* and *npa1l1–2/+* mutants contained four distinct classes of developmentally impaired embryos, including those arrested at the globular (3.50 and 5.67, respectively), heart (1.00 and 2.50), torpedo (9.00 and 7.17), and cotyledon (0.50 and 0.33) stages (Table 2). The combined frequency of these defective embryos was approximately 25% in both heterozygous mutants, consistent with the expected ratio of homozygous progeny (Table 1). These results indicate that *npa1l1* homozygous mutants exhibit varying degrees of developmental delay or arrest, starting at the globular stage.

**Table 2:** Distribution of embryo development stages in the WT and mutants in 7 DAP siliques

Parents	DAP	Nts/Nsi	Nas	DG	DH	H	DT	T	DC	C	Percentage aborted
WT	7	340/6	56.67	0.17	—	0.17	—	0.66	0.17	55.50	0.60%
*npa1l1‐1/+*	7	348/6	58.00	3.50	1.00	—	9.00	—	0.50	44.00	24.14%
*npa1l1‐2/+*	7	371/6	61.83	5.67	2.50	—	7.17	—	0.33	46.16	25.34%

*Notes*: The siliques at 7 days after pollination (DAP) were continuously marked. The average number of embryos at different developmental stages from six siliques is presented. Percentage aborted = (DG + DH + DT + DC)/Nas × 100%. Red numbers indicate the number of defective embryos.

Abbreviations: C, Cotyledon; DAP, Day after pollination; DC, Defective cotyledon; DG, Defective globular; DH, Defective heart; DT, Defective torpedo; H, Heart; Nas, Average number of ovules per silique; Nts/Nsi, Number of total ovules/number of siliques; T, Torpedo; WT, Wild type.

To investigate whether the defective embryo development in *npa1l1* mutants was associated with endosperm, we examined the endosperm development in WT, *npa1l1–1/+*, and *npa1l1–2/+* plants. At the zygote and late globular embryo stages, embryos of both WT and mutant plants were surrounded by free endosperm nuclei, with no obvious differences between WT and mutant plants (Figure S7a–l). At the heart embryo stage, endosperm cellularization occurred in WT plants (Figure S7m,n). In contrast, in the mutants, endosperm failed to initiate cellularization, and the embryo is surrounded by fewer uncellularized endosperm nuclei (Figure S7q,r,u,v). At the torpedo embryo stage, endosperm cells of WT underwent persistent proliferation and further cellularization, with numerous small endosperm nuclei (Figure S7o,p). In the mutants, however, most endosperm nuclei were degraded, and the seeds began to shrink (Figure S7s,t,w,x). These results indicate that, starting from the heart embryo stage, the mutants exhibit asynchronous development between embryos and endosperm, accompanied by impaired endosperm cellularization and degeneration.

In summary, *npa1l1* mutant embryos exhibit defects in cell division orientation and cytokinesis starting from the late globular stage, and defective endosperm cellularization and endosperm degeneration occur at the heart embryo stage.

### Plant Npa1L1 protein is involved in ribosome biogenesis

**Figure 3:**
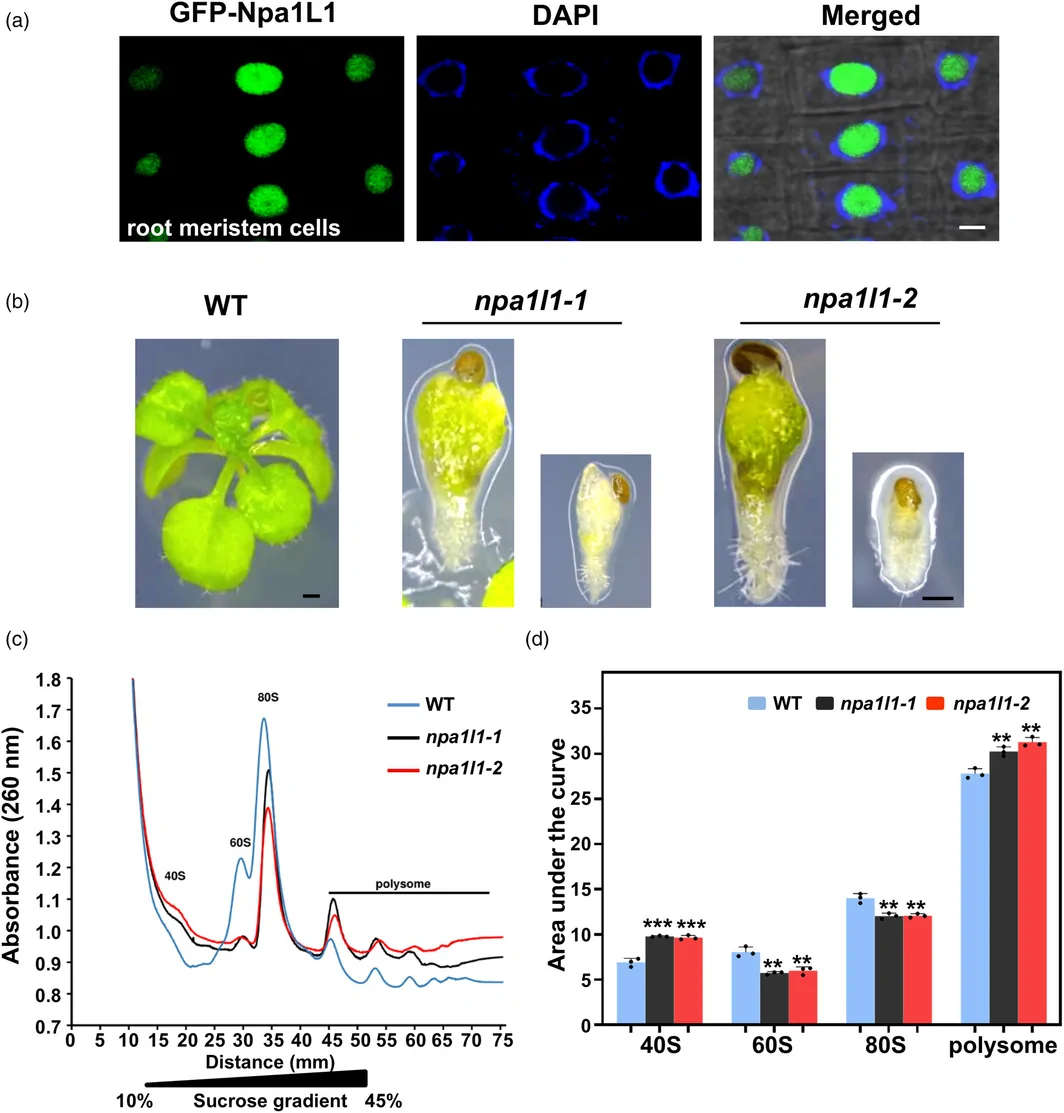
Npa1L1 localizes to the nucleolus and polyribosome profiles are aberrant in *npa1l1* mutants. (a) Npa1L1 localizes to the nucleolus of root tip meristem cells from 10‐day‐old seedlings in complemented transgenic plants expressing pNpa1L1::GFP‐Npa1L1. The nucleoplasm was stained with 4′,6‐diamidino‐2‐phenylindole (DAPI). Scale bar = 3 μm. (b) WT and *npa1l1* seedlings grown for 14 days, respectively. An individual scale bar is provided for WT, and a shared scale bar is used for *npa1l1‐1* and *npa1l1‐2*; all scale bars = 0.5 mm. (c) Polysome profiles of WT, *npa1l1‐1*, and *npa1l1‐2* seedlings. Ribosomal components (40S small subunits, 60S large subunits, 80S monosomes, and polysome fractions) were separated by sucrose density gradient centrifugation. Representative absorbance curves at 260 nm are shown; the profiles represent the average of three independent biological replicates. (d) Quantification of the area under the curve (AUC) for 40S, 60S, 80S, and polysome fractions. Values represent the mean ± SD of three biological replicates. Significant differences between WT and mutant lines were determined by Student's *t*‐test (**P < 0.01, ***P < 0.001).

The yeast Npa1p is a nucleolar protein and participates in ribosome assembly (Dez et al., 2004). To determine whether plant Npa1L1 played a similar role, we began by examining its subcellular localization. We used a pNpa1L1::GFP‐Npa1L1/*npa1l1* complementation line, which fully complemented the mutant phenotype (Figure 1b–d; Figure S5d), confirming the functionality of the GFP fusion protein. Confocal imaging of root tip meristems revealed that the GFP‐Npa1L1 signal was detected in the nucleus (Figure 3a). To precisely define the intranuclear localization of GFP‐Npa1L1, we performed co‐staining with 4′,6‐diamidino‐2‐phenylindole (DAPI), which strongly labels DNA‐rich regions such as the nucleoplasm but does not stain the RNA‐rich nucleolus (Ross et al., 1996). The GFP fluorescence signal did not colocalize with DAPI‐stained areas (Figure 3a), indicating that Npa1L1 was likely a nucleolar protein—a finding consistent with a potential function in ribosome biogenesis.

To investigate whether mutations in *Npa1L1* affected ribosome biogenesis, we attempted to analyze WT and *npa1l1* homozygous mutants. However, no homozygous *npa1l1* plants reached maturity (Table 1). Notably, seeds of WT and *npa1l1/+* heterozygous mutants were germinated and cultured on medium for 14 days. While WT plants had developed multiple true leaves at this time point, some seeds failed to germinate, and abnormal seedlings were observed in *npa1l1/+* (Figure 3b). These mutant seedlings exhibited severe developmental defects, including small size and missing or malformed cotyledons, and failed to develop further (Figure 3b). Genotypic analysis confirmed that these arrested seedlings were homozygous for *npa1l1‐1* or *npa1l1‐2* (Figure S5c). These homozygous mutant seedlings were subsequently used as material for analyzing ribosomal‐related experiments.

To assess whether the Npa1L1 mutation affected ribosome biogenesis, we performed polysome profile analysis. Cell extracts from WT and *npa1l1* mutant 14‐day seedlings were separated via sucrose density gradient centrifugation, and ribosomal subunits (40S and 60S), monosomes (80S), and polysomes were quantified by monitoring absorbance at 260 nm. The area under the curve (AUC) corresponding to 40S, 60S, 80S, and polysome fractions was calculated. The *npa1l1* mutant showed higher levels of 40S subunits, along with reduced 60S subunits and 80S monosomes, whereas polysome abundance increased compared with the WT (Figure 3c,d). Thus, the loss of *Npa1L1* gene affected ribosome biogenesis.

### Npa1L1 is required for pre‐rRNA processing during ribosome biogenesis

**Figure 4:**
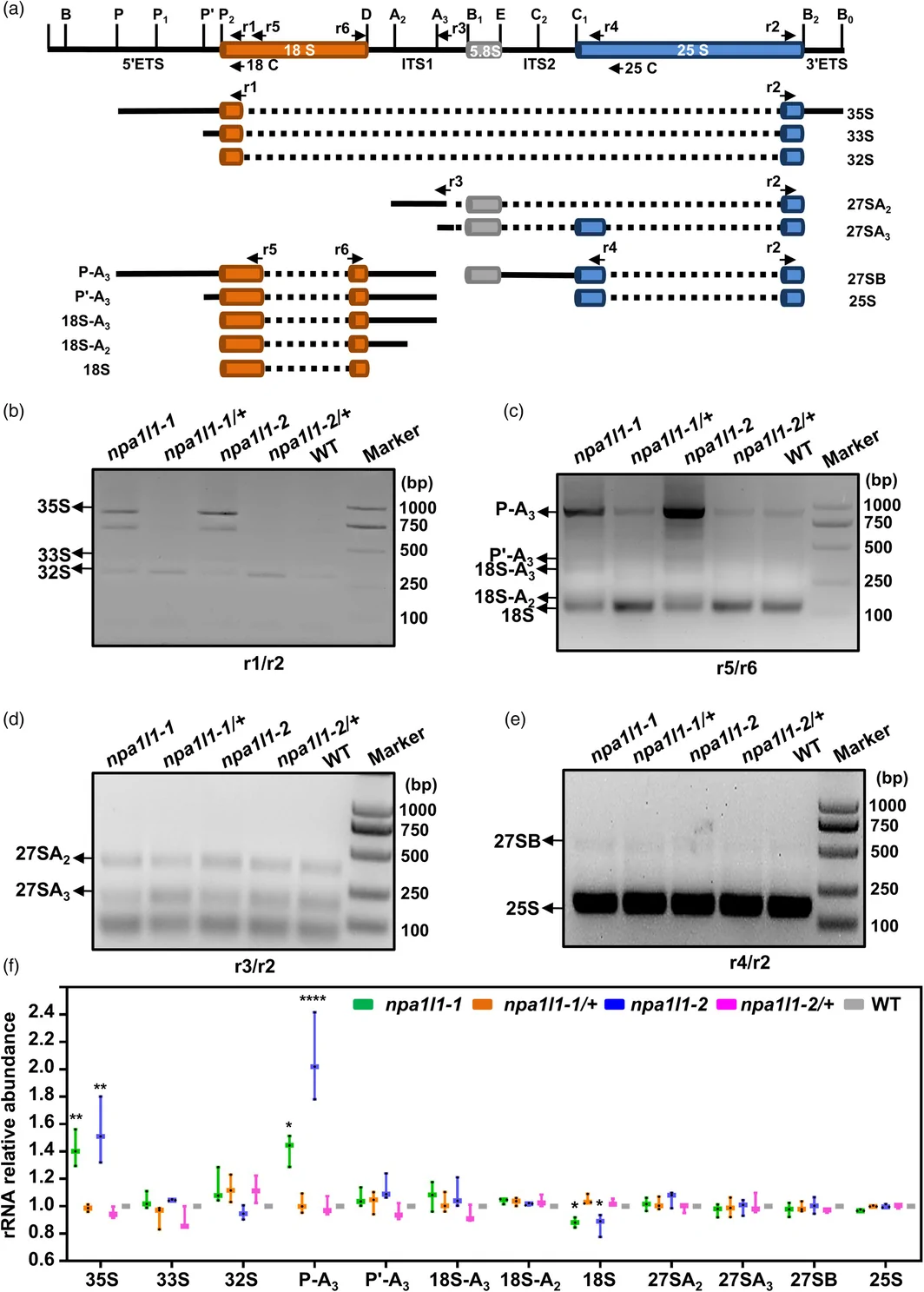
Circular RT‐PCR reveals defective pre‐rRNA processing in *npa1l1* mutants. (a) Schematic diagram showing the positions of primers and pre‐rRNA processing intermediates detected by circular RT‐PCR. Primers 18c and 25c were used for reverse transcription. (b) Accumulation of 35S pre‐rRNA was observed in *npa1l1‐1* using primer set r1/r2. (c) Accumulation of P‐A_3_ intermediates, along with reduced levels of mature 18S rRNA, was detected in *npa1l1‐1* using primer set r5/r6. (d) No significant differences in 27SA_2_ and 27SA_3_ pre‐rRNA levels were observed among WT, *npa1l1*, and *npa1l1/+* plants, as detected using primer set r3/r2. (e) No obvious alterations in 27SB pre‐rRNA and mature 25S rRNA were observed among WT, *npa1l1*, and *npa1l1/+* plants, as detected using primer set r4/r2. (f) The rRNA relative levels of *npa1l1* and *npa1l1/+* mutants were obtained by normalizing to the WT control. All data were obtained from three biological replicates, with error bars indicating standard deviation (SD). Significant differences between WT and mutant lines were determined by one‐way ANOVA (*P < 0.05, **P < 0.01, ****P < 0.0001).

In eukaryotic ribosome biogenesis, a common precursor rRNA (pre‐rRNA) is processed to yield the mature 18S, 5.8S, and 25S/28S rRNAs, which are essential structural components of the 40S and 60S ribosomal subunits, respectively (Klinge & Woolford, 2019; Saez‐Vasquez & Delseny, 2019). To determine whether Npa1L1 was involved in pre‐rRNA processing in Arabidopsis, we performed circular RT‐PCR (cRT‐PCR) analysis. Total RNA was circularized with T4 RNA ligase and reverse transcribed with gene‐specific primer 18c or 25c, and the resulting cDNA was amplified with specific primer pairs to examine key pre‐rRNA processing intermediates (Figure 4a). Amplification with primers r1/r2 revealed strong accumulation of the 35S pre‐rRNA and no obvious difference in 33S and 32S pre‐rRNAs in *npa1l1* homozygous mutants compared to WT plants (Figure 4b,f). Using primers r5/r6 to detect the 18S rRNA precursor processing, we observed a significant accumulation of early 18S rRNA precursors (P‐A_3_), increased but not significant accumulation of 18S rRNA precursors (P′‐A_3_, 18S‐A_3_, 18S‐A_2_), and obvious reduction of mature 18S rRNA in *npa1l1* mutants (Figure 4c,f). We next examined processing of the large subunit rRNA using primers r3/r2 and r4/r2, which detected 27S‐A_2_, 27S‐A_3_, 27SB pre‐rRNAs, and mature 25S rRNA. The abundance of these rRNAs was comparable between WT and *npa1l1* plants (Figure 4d–f). Moreover, no obvious changes in pre‐rRNA processing were detected between heterozygous *npa1l1/+* and WT (Figure 4b–f).

Collectively, mutation of Npa1L1 causes obvious accumulation of 35S and P‐A_3_ pre‐rRNAs, along with reduced abundance of mature 18S rRNA, while 27S rRNA precursor processing is not significantly affected. Thus, Npa1L1 mutation significantly affects rRNA processing.

**Figure 5:**
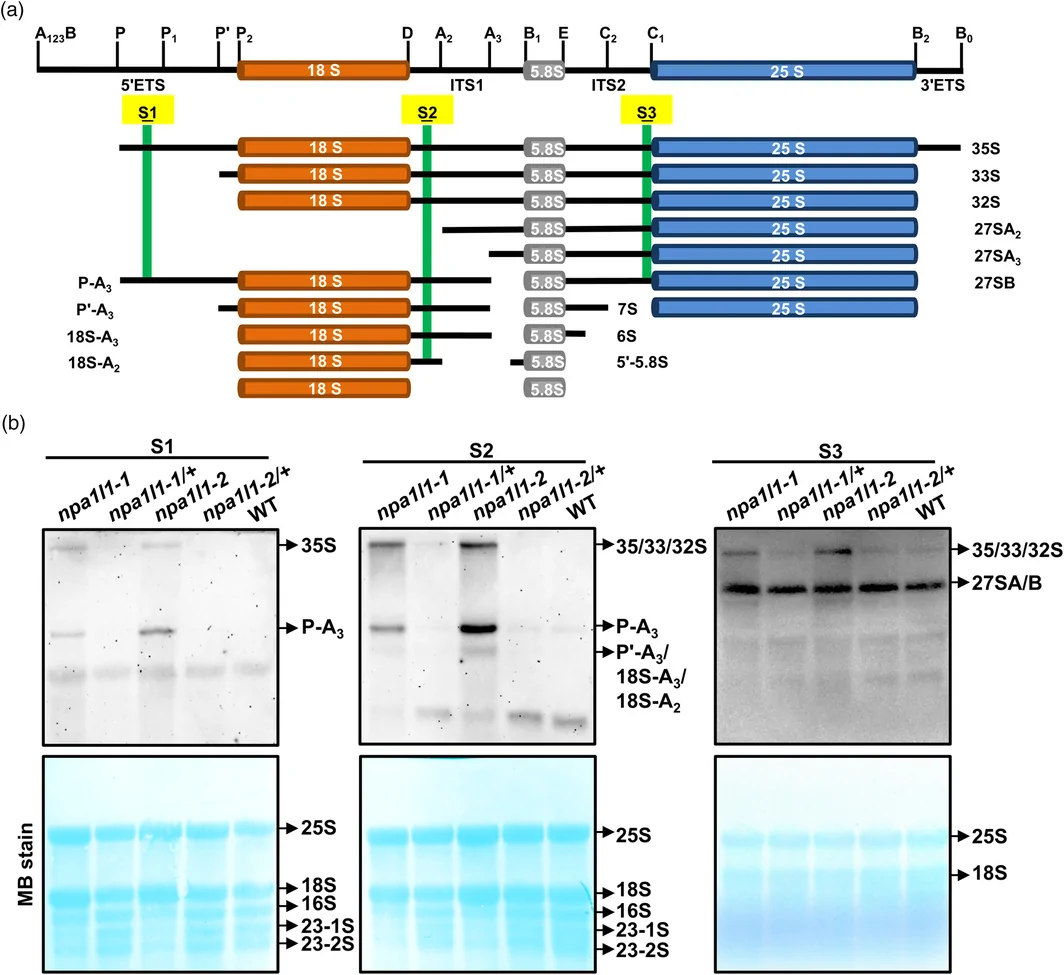
Pre‐rRNA processing is perturbed in *npa1l1* mutants as revealed by northern blot analysis. (a) Diagram illustrating the cleavage sites and various pre‐rRNA processing intermediates. The green frame indicates the pre‐rRNA processing intermediates detected by northern blot using specific probes S1, S2, and S3. ETS: external transcribed spacer; ITS: internal transcribed spacer. (b) Total RNA was separated on 1.2% formaldehyde‐agarose gels, transferred to a nylon membrane, and hybridized with probes S1, S2, and S3. Northern blot analysis revealed aberrant accumulation of 35/33/32S pre‐rRNAs and 18S rRNA precursors (P‐A_3_ and P′‐A_3_/18S‐A_3_/18S‐A_2_) in *npa1l1* mutants when detected with probes S1, S2, and S3. In contrast, no significant difference in 27S rRNA precursors (27SA/B) was observed between the WT and the mutant when probed with S3. Methylene blue staining of the membrane is shown as a loading control. The 16S, 23‐1S, and 23‐2S bands correspond to chloroplast rRNAs.

We further employed northern blot analysis to assess the impact of *Npa1L1* mutation on pre‐rRNA processing. Three oligonucleotide probes (S1, S2, and S3) were designed to target the 5′ETS, ITS1, and ITS2 regions to detect distinct pre‐rRNA intermediates (Figure 5a). The S1 probe mainly detects 35S and P‐A_3_ pre‐rRNAs, while the S2 probe recognizes 35S/33S/32S pre‐rRNAs and multiple 18S rRNA precursors, including P‐A_3_, P′‐A_3_, 18S‐A_3_, and 18S‐A_2_. The S3 probe hybridizes to 35S/33S/32S pre‐rRNA as well as 27SA and 27SB pre‐rRNAs (Figure 5a). Hybridization with the S1 probe revealed pronounced accumulation of both 35S and P‐A_3_ pre‐rRNAs in *npa1l1* homozygous mutants (Figure 5b). Using the S2 probe, we observed significant accumulation of 35S/33S/32S, P‐A_3_, and P′‐A_3_/18S‐A_3_/18S‐A_2_ pre‐rRNA intermediates in *npa1l1* homozygous mutants (Figure 5b). Hybridizing with the S3 probe revealed obvious accumulation of 35S/33S/32S pre‐rRNA, while the abundance of 27SA and 27SB intermediates in *npa1l1* homozygous mutants was comparable to WT (Figure 5b). Additionally, *npa1l1/+* heterozygous plants showed no obvious alterations in pre‐rRNA abundance compared to the WT.

These northern blot results are consistent with our cRT‐PCR observations, providing further support for the accumulation of 35S and P‐A_3_ pre‐rRNA intermediates in *npa1l1* mutants. Together, mutation of *Npa1L1* leads to 18S rRNA precursor processing defects and affects ribosome biogenesis.

### Npa1L1 physically interacts with the DEAD‐box RNA helicases RH7/RH27 and GTPase NSN1, collectively contributing to pre‐rRNA processing

In yeast, Npa1p forms a complex with nucleolar pre‐ribosomal‐associated protein 2 (Npa2p), nucleolar protein 8 (Nop8p), DEAD‐box RNA‐binding protein 6 (Dbp6p), and ribosome assembly protein 3 (Rsa3p), acting as a core scaffold component to facilitate early 60S pre‐ribosomal particle assembly and 27S rRNA precursor processing (Joret et al., 2018; Rosado et al., 2007). To examine whether Arabidopsis Npa1L1 functions in a similar protein complex during pre‐rRNA maturation, we screened its putative interacting partners based on known interactors of yeast and human Npa1 homologs. Human DEAD‐box RNA helicase 21 (DDX21), yeast Helicase Associated with Set1 (Has1p), and Nuclear GTPase 1 (Nug1p) are putative interacting partners of Npa1 and participate in pre‐rRNA processing and ribosome biogenesis (Bassler et al., 2001; Calo et al., 2015; Dembowski et al., 2013; Dez et al., 2004; Emery et al., 2004; Sekiguchi et al., 2006). We further identified their respective Arabidopsis orthologs: human DDX21 corresponds to RNA Helicase 7 (RH7), yeast Has1p corresponds to RNA Helicase 27 (RH27), and yeast Nug1p corresponds to Nucleostemin‐like 1 (NSN1). All three Arabidopsis proteins localize to the nucleolus and are involved in plant embryonic development (Hou et al., 2021; Huang et al., 2016; Jeon et al., 2015; Liu et al., 2016; Wang et al., 2012). RH7 and RH27 are ATP‐dependent DEAD‐box RNA helicases, and NSN1 is a GTPase (Hou et al., 2021; Huang et al., 2016; Jeon et al., 2015; Liu et al., 2016; Wang et al., 2012). Notably, RH7 has been previously reported to participate in pre‐rRNA processing (Huang et al., 2016; Liu et al., 2016).

**Figure 6:**
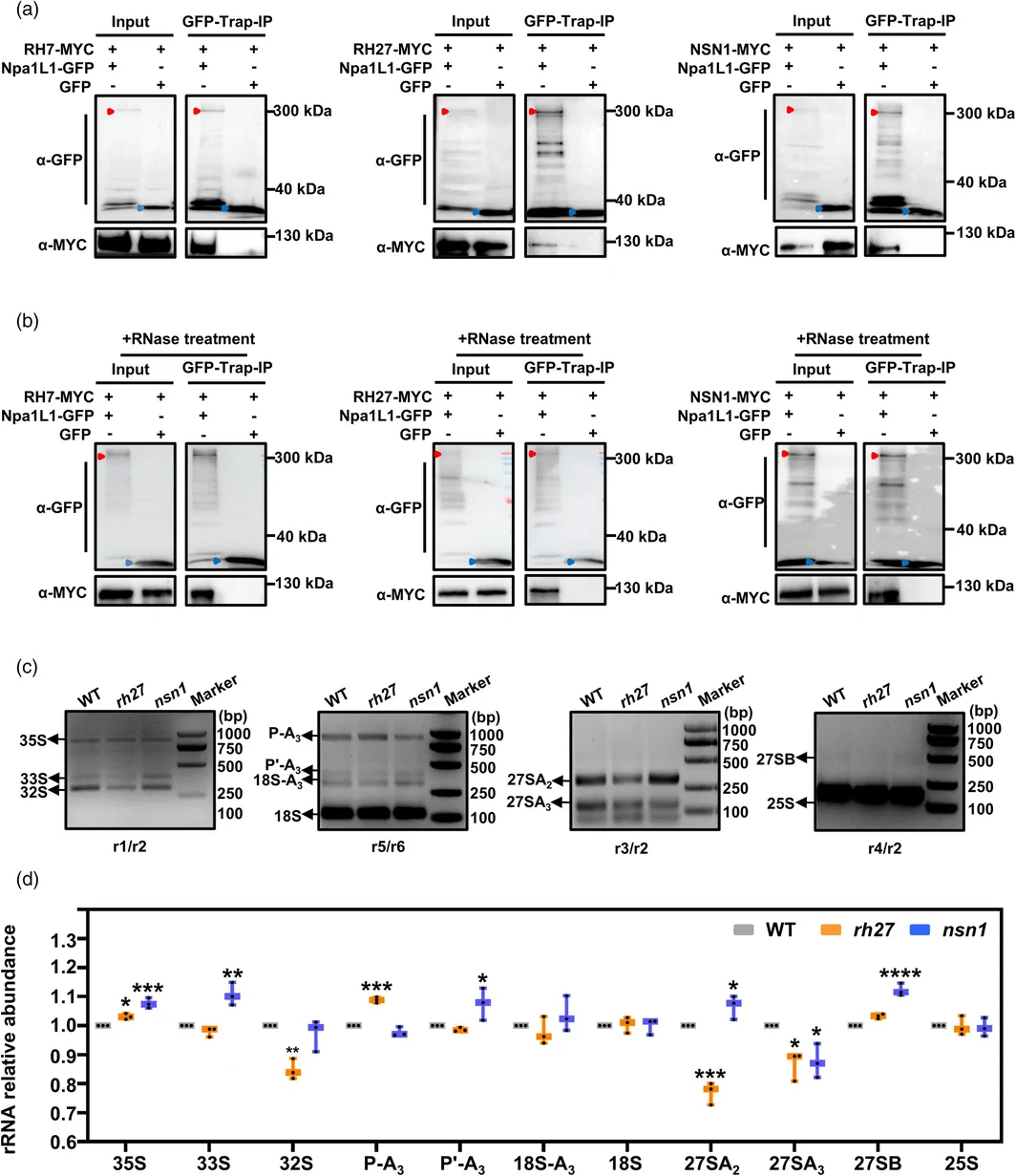
Npa1L1 interacts with RH7, RH27, and NSN1 *in vivo*, and circular RT‐PCR analysis reveals altered pre‐rRNA processing in *rh27* and *nsn1* mutants. (a) Co‐immunoprecipitation (Co‐IP) assays were performed to investigate the interaction between GFP‐tagged Npa1L1 and MYC‐tagged RH7, RH27, and NSN1 in Arabidopsis. RH7‐MYC, RH27‐MYC, and NSN1‐MYC were successfully co‐precipitated with Npa1L1‐GFP, whereas no binding was detected in the control groups (empty GFP vector alone and MYC‐tagged RH7, RH27, and NSN1 alone). Red arrowheads indicate the Npa1L1‐GFP protein, and blue arrowheads indicate the GFP protein. (b) Co‐IP assays with RNase treatment were conducted to further verify the interaction between GFP‐tagged Npa1L1 and MYC‐tagged RH7, RH27, and NSN1 in Arabidopsis. All lysates were treated with RNase A prior to IP. Consistent with the results in (a), RH7‐MYC, RH27‐MYC, and NSN1‐MYC were co‐precipitated by Npa1L1‐GFP, while no specific interaction was observed in the control samples. Red arrowheads mark the Npa1L1‐GFP protein, and blue arrowheads mark the GFP protein. (c) In the *rh27* mutant, accumulation of 35S and P‐A_3_ pre‐rRNA intermediates was observed, accompanied by decreased levels of 32S, 27SA_2_, and 27SA_3_ pre‐rRNAs. In the *nsn1* mutant, 35S, 33S, P′‐A_3_, 27SA_2_, and 27SB pre‐rRNAs were accumulated, whereas 27SA_3_ pre‐rRNA was downregulated. No significant differences in the abundance of 18S and 25S rRNAs were observed among WT, *rh27*, and *nsn1*. (d) The relative levels of *rh27* and *nsn1* mutants were normalized to the WT control. All data were obtained from three biological replicates, with error bars indicating standard deviation (SD). Significant differences between WT and mutant lines were determined by one‐way ANOVA (*P < 0.05, **P < 0.01, ***P < 0.001, ****P < 0.0001).

To test whether Npa1L1 interacted with RH7, RH27, and NSN1 *in vivo*, we performed co‐immunoprecipitation (Co‐IP) assays. Transgenic plants were generated expressing N‐terminal GFP‐tagged Npa1L1 under the 35S promoter, as well as C‐terminal 6 × MYC‐tagged versions of RH7, RH27, and NSN1. These lines were subsequently crossed to obtain plants co‐expressing GFP‐Npa1L1 with each MYC‐tagged candidate. Immunoprecipitation using anti‐GFP antibodies revealed that RH7‐MYC, RH27‐MYC, and NSN1‐MYC each co‐precipitated with GFP‐Npa1L1, but not with GFP alone (Figure 6a). These results demonstrate that Npa1L1 physically interacts with the three proteins.

Since rRNA can serve as a structural scaffold to mediate interactions among ribosome‐associated proteins (Giuliano & Engl, 2021), we next performed Co‐IP assays after degrading rRNA with ribonuclease (RNase) to verify whether the interactions between Npa1L1, RH7, RH27, and NSN1 observed in this study are RNA‐dependent (Figure 6b). The results showed that Npa1L1 still interacted with RH7, RH27, and NSN1 in the absence of RNA, indicating that these interactions are RNA‐independent (Figure 6b).

Previous studies have shown that, similar to *npa1l1* mutant, loss of function of RH7 leads to the accumulation of 35S pre‐rRNA and multiple 18S rRNA precursors, including P‐A_3_, P′‐A_3_, 18S‐A_3_, and 18S‐A_2_ (Huang et al., 2016; Liu et al., 2016). To determine whether NSN1 and RH27 also participated in pre‐rRNA maturation, we analyzed their pre‐rRNA processing in homozygous mutants of *rh27* and *nsn1* using cRT‐PCR. Similar to the *npa1l1* mutant, *rh27* and *nsn1* showed accumulation of the 35S pre‐rRNA. Regarding 18S rRNA precursor processing, *RH27* deficiency caused a marked accumulation of the P‐A_3_ pre‐rRNA, a phenotype also observed in the *npa1l1* mutant (Figure 6c,d). Mutation in *NSN1* resulted in a slight increase in the P′‐A_3_ pre‐rRNA level, but unlike *npa1l1*, neither the *rh27* nor the *nsn1* mutation affected the steady‐state level of mature 18S rRNA. With respect to 27S rRNA precursor processing, the two mutants exhibited effects on the abundance of 27S pre‐rRNA intermediates that differed from those seen in the *npa1l1* mutant. However, similar to *npa1l1*, no significant changes in the accumulation of mature 25S rRNA were observed in either mutant (Figure 6c,d). Collectively, these results suggested that loss of function of *RH7* or *RH27* causes defects in 18S rRNA precursor processing that are more similar to those of the *npa1l1* mutant.

In conclusion, our findings demonstrate that Npa1L1 physically associates with the DEAD‐box RNA helicases RH7 and RH27, as well as the GTPase NSN1, and that these interactions contribute collectively to ribosomal RNA precursor processing in Arabidopsis.

## DISCUSSION

rRNA is a key component of ribosomes, and its formation relies on the complex pre‐rRNA processing, which requires the involvement of a large number of RBFs (Palm et al., 2019; Weis et al., 2015). Therefore, identifying the functions of key RBFs involved in ribosome pre‐rRNA processing is of great significance. Pre‐rRNA processing in plants is more complex than in yeast and humans, but current research on RBFs involved in pre‐rRNA processing in plants is very limited (Weis et al., 2015). This study suggests that Arabidopsis Npa1L1, a previously uncharacterized protein, may act as a ribosome biogenesis factor involved in 18S rRNA precursor processing and ribosomal subunit assembly (Figures 3c,d, 4a–f, 5a,b).

### Potential functions of Npa1L1 in ribosomal pre‐rRNA processing

We carried out ribosome profiling (Figure 3c,d), cRT‐PCR (Figure 4a–f), and northern blot analysis to detect ribosome biogenesis and pre‐rRNA processing (Figure 5a,b). The ribosome profiling results show that, compared to WT, the *npa1l1* mutant exhibits reduced abundance of 60S subunits and 80S monosomes, together with increased levels of 40S subunits and polysomes (Figure 3c,d). The cRT‐PCR and northern blot data reveal a dramatic accumulation of 35S and P‐A_3_ pre‐rRNAs, along with a mild accumulation of P′‐A_3_ and 18S‐A_2_/A_3_ pre‐rRNAs; mature 18S rRNA is significantly decreased, whereas 27SA/B pre‐rRNA and mature 25S rRNA remain largely unchanged (Figures 4a–f, 5a,b). Together, mutation of *Npa1L1* leads to defective processing of 18S rRNA precursors and affects ribosome biogenesis. Our findings indicate that Npa1L1, an important plant ribosome biogenesis factor, plays essential roles in rRNA processing and ribosome biogenesis.

A limitation of this study is the absence of an inducible *Npa1L1* knockdown system, which precluded analysis of its immediate effects, together with the lack of protein–RNA interaction data that could identify direct RNA targets and reveal the primary defects in pre‐rRNA processing. Thus, current analysis cannot distinguish whether the observed 18S rRNA precursors accumulation and reduced mature 18S rRNA are direct effects of the *Npa1L1* mutation or indirect consequences of developmental arrest.

Yeast Npa1p is well characterized as an essential scaffold that directly binds 27SA_2_ pre‐rRNA to stabilize the earliest pre‐60S ribosomal particle (Choque et al., 2018). Regarding whether Arabidopsis Npa1L1 shares the same molecular function as yeast Npa1p, we offer the following inferences based on our current data. One possibility, therefore, is that Arabidopsis Npa1L1 and yeast Npa1p have conserved functions, both contributing primarily to 60S large subunit biogenesis. In our *npa1l1* mutant, we observe substantial accumulation of P‐A_3_ pre‐rRNA. Similarly, deletion of yeast Npa1p leads to marked accumulation of the corresponding 23S pre‐rRNA (Dez et al., 2004; Rosado & De La Cruz, 2004). Moreover, loss of Npa1L1 in Arabidopsis and loss of Npa1p in yeast both result in reduced mature 18S rRNA levels. Importantly, defects in 60S subunit biogenesis are known to cause secondary accumulation of 18S rRNA precursors; for example, mutation of the Arabidopsis 60S‐associated ribosome biogenesis factor Large Subunit GTPase 1–2 (LSG1‐2) leads to massive accumulation of 18S rRNA precursors (Missbach et al., 2014). Thus, the strong 18S rRNA precursor processing defects in the *npa1l1* mutant could be an indirect consequence of impaired 60S function, consistent with a conserved role for Npa1 in large subunit biogenesis. Notably, we do not detect obvious defects in 27S rRNA precursors processing in the *npa1l1* mutant. This is likely explained by the presence of a second paralog in Arabidopsis, Npa1L2, which shares 66.01% amino acid sequence identity with Npa1L1 (Figure S3a). Functional redundancy between the two proteins may allow Npa1L2 to compensate for 27S rRNA precursor processing defects caused by loss of Npa1L1. Future work will investigate the biological role of Npa1L2 to test this hypothesis directly.

A second possibility is that Arabidopsis Npa1L1 has functionally diverged from its yeast counterpart. First, two distinct rRNA processing pathways exist in Arabidopsis: the ITS1‐first (major) pathway and the 5′ETS‐first (minor) pathway. In the major ITS1‐first pathway, cleavage occurs at the A_3_ site (equivalent to yeast A_3_). In yeast, however, processing initiates preferentially within the 5′ETS region, resembling the 5′ETS‐first pathway in Arabidopsis. The 23S pre‐rRNA generated by cleavage at the yeast A_3_ site is considered an aberrant intermediate and is rapidly degraded (Choque et al., 2018; Klinge & Woolford, 2019). In the *npa1l1* mutant, we observe accumulation of P‐A_3_, P′‐A_3_, and 18S‐A_2_/A_3_ pre‐rRNAs, which likely contributes to the reduction in mature 18S rRNA levels. By contrast, in yeast, the aberrantly accumulated 23S pre‐rRNA is degraded and therefore does not substantially affect mature 18S rRNA production (Figure S9). This evolutionary divergence in rRNA cleavage pathways may reflect functional diversification of homologous proteins.

Second, although both Npa1L1 and yeast Npa1p contain conserved Npa1 and NopRA1 domains, the amino acid sequence identity of the Npa1 domain is only 17.72%, and that of the NopRA1 domain is only 15.88% (Figure S3b,c), suggesting that these key domains have diverged during evolution. Furthermore, the molecular mass of Arabidopsis Npa1L1 is 289 kDa, considerably larger than that of yeast Npa1p (203 kDa) (Figure S1a), and the two proteins show marked differences in sequence length. Such sequence variations may alter protein conformation, reduce substrate binding capacity, and reshape interaction patterns with other factors.

Third, the pronounced accumulation of P‐A_3_ pre‐rRNA in the *npa1l1* mutant may indicate delayed or defective cleavage at the P′ site. The P′ site (equivalent to the yeast A_0_ site) is a key cleavage site for the 5′ETS‐first processing pathway in Arabidopsis, and impaired cleavage at this site likely accounts for the observed accumulation of 35S pre‐rRNA. We therefore hypothesize that Arabidopsis Npa1L1 plays an important role in P‐A_3_ pre‐rRNA processing (Figure S8).

It remains unclear which of these two scenarios represents the true function of Npa1L1. Future studies using inducible knockdown systems and direct RNA‐binding assays are required to define Npa1L1's primary function and precise molecular role in rRNA processing.

Unexpectedly, the Arabidopsis *npa1l1* mutant exhibited reduced levels of mature 18S rRNA yet increased abundance of the 40S ribosomal subunit. The 25S rRNA content remained unchanged, while the abundance of the 60S ribosomal subunit decreased (Figures 3c,d, 4a–f). Several previous studies have reported such discrepancies between rRNA levels and ribosomal subunit abundance. Mutations in the Arabidopsis ribosome biogenesis factor LSG1‐2 lead to decreased abundance of the 60S subunit without altering 25S rRNA levels (Missbach et al., 2014). In addition, one plausible explanation is that immature pre‐40S subunits are enriched in the 40S peak during polysome profiling. For instance, pre‐40S particles containing immature 18S rRNA precursors (P‐A_3_, P′‐A_3_, 18S‐A_3_, and 18S‐A_2_) accumulate substantially in the 40S fraction in the Arabidopsis *hot3* mutant (Hang et al., 2023). Similarly, pre‐40S particles carrying 20S pre‐rRNA are enriched in the 40S fraction in the yeast *nob1* mutant (Fatica et al., 2003). The elevated 40S subunit abundance in the *npa1l1* mutant may result from increased P‐A_3_ levels, which causes accumulation of immature pre‐40S particles containing P‐A_3_ pre‐rRNA. However, we currently lack direct validation via sucrose gradient fractionation followed by northern blotting targeting the P‐A_3_ fragment, and therefore, it remains unclear whether P‐A_3_ pre‐rRNA accumulates in the pre‐40S fraction in the *npa1l1* mutant.

Ribosome biogenesis is fundamental for cellular protein synthesis (Woolford Jr. & Baserga, 2013). When the efficiency of ribosome biogenesis decreases and the pool of functional ribosomes is reduced, plants may upregulate rRNA transcription or ribosomal subunit synthesis through feedback pathways in an attempt to compensate for the shortage of ribosomes (Chandrasekhara et al., 2021). Therefore, another possibility is that defective pre‐rRNA cleavage in the *npa1l1* mutant impedes mature 18S rRNA production, while simultaneously triggering a compensatory upregulation of pre‐40S synthesis through a ribosome biogenesis feedback pathway. This could account for the paradoxical phenotype of reduced mature 18S rRNA despite increased 40S abundance in the *npa1l1* mutant.

Furthermore, rRNA processing and ribosome assembly are highly precise and energy‐consuming processes (Tomecki et al., 2017). To ensure that only functionally intact ribosomes participate in protein synthesis, plants have evolved quality control systems that monitor each step from processing to mature assembly and promptly eliminate defective or excess rRNA intermediates (Chen et al., 2026; Parker & Karbstein, 2023). Accordingly, it is also possible that some assembled 40S particles contain incompletely processed or defective 18S rRNA and may be subjected to rRNA quality control surveillance, resulting in reduced mature 18S rRNA coupled with increased 40S abundance. Clearly, further experiments are required to test these hypotheses.

### Distinct and cooperative roles of Npa1L1, RH7, RH27, and NSN1 in pre‐rRNA processing and ribosome assembly

Yeast Npa1p functions as a scaffold protein that recruits other factors to form complexes and participates in 27S rRNA precursor processing and 60S large subunit biogenesis (Joret et al., 2018). In this study, Co‐IP assays confirmed that Npa1L1 physically interacts with RH7, RH27, and NSN1 in an RNA‐independent manner (Figure 6a,b). Notably, all three interacting proteins have been reported to localize to the nucleolus (Hou et al., 2021; Jeon et al., 2015; Liu et al., 2016), and mutations in each of these genes cause severe embryonic defects (Hou et al., 2021; Huang et al., 2016; Wang et al., 2012), which are reminiscent of the *npa1l1* mutant. Collectively, these observations revealed that Npa1L1 and its interacting partners may function in a common pathway to coordinate ribosome biogenesis. Based on our experimental results in pre‐rRNA processing and the reported functions of RH7, RH27, and NSN1, together with their yeast and human orthologs, we propose possible mechanisms for how these interactions may contribute to pre‐rRNA processing and ribosome assembly.

In the Arabidopsis *rh7* mutant, accumulation of both 35S and P‐A_3_ pre‐rRNAs was observed (Huang et al., 2016; Liu et al., 2016), a phenotype similar to that of *npa1l1*. Although the level of mature 18S rRNA is not significantly altered in the *rh7* mutant, its resistance to streptomycin suggests a role for RH7 in 40S small subunit assembly (Huang et al., 2016; Liu et al., 2016). Although plant RH7 has no direct ortholog in yeast, its human counterpart DDX21 has been reported to directly bind 18S rRNA and is critical for 40S ribosomal subunit maturation (Calo et al., 2015; Sloan et al., 2015). Arabidopsis RH7 belongs to the DEAD‐box RNA helicase family, members of which function in ribosome biogenesis by unwinding rRNA secondary structures to expose processing sites or by facilitating proper rRNA folding (Karbstein, 2022; Mitterer and Pertschy, 2022). Notably, yeast Npa1p has been shown to recruit the DEAD‐box RNA helicase Dbp6p to 60S particles, where Dbp6p utilizes ATP hydrolysis to remodel RNA structures or modulate RNA–protein interactions, a process essential for proper early 25S rRNA folding (Joret et al., 2018; Khreiss et al., 2023). If Npa1L1 functions primarily in 18S rRNA precursor processing and 40S subunit assembly, it may achieve this by acting as a scaffold to recruit RH7, whose helicase activity may facilitate 18S rRNA maturation.

In contrast, Arabidopsis DEAD‐box RNA helicase RH27 has been identified as a component of the microprocessor complex involved in miRNA processing, with no reported function in rRNA processing (Hou et al., 2021). Our data revealed that the *rh27* mutant accumulated 35S and P‐A_3_ pre‐rRNAs and also exhibited reduced levels of 27SA_2_/A_3_ pre‐rRNA (Figure 6c,d), suggesting that RH27 may participate in 18S and 27S rRNA precursor processing. Nevertheless, these results are insufficient to define the precise role of RH27 in pre‐rRNA processing. Evolutionary conservation provides some clues: the yeast ortholog of RH27, Has1p, has been reported to directly bind both 18S and 25S rRNAs and is involved in both 40S and 60S subunit biogenesis (Dembowski et al., 2013; Emery et al., 2004; Gnanasundram et al., 2019). Moreover, yeast Has1p was identified as a candidate interactor of Npa1p in co‐immunoprecipitation coupled with mass spectrometry, and the rRNA processing defects observed in *has1* and *npa1* mutants are highly similar (Dembowski et al., 2013; Dez et al., 2004; Emery et al., 2004). Thus, it is plausible that plant RH27 is involved in both 40S and 60S ribosomal subunit biogenesis. Therefore, regardless of whether Npa1L1 participates in 40S or 60S ribosomal subunit biogenesis, it may similarly act as a scaffold protein to recruit the DEAD‐box RNA helicase RH27, thereby coordinately facilitating ribosome biogenesis.

Unlike RH7 and RH27, the function of NSN1 is more clearly linked to the 60S large subunit biogenesis. Our data show that the *nsn1* mutant exhibited elevated 35S pre‐rRNA levels, mild increases in P′‐A_3_ and 27SA_2_ pre‐rRNA levels, and decreased levels of 27SA_3_ pre‐rRNA (Figure 6c,d). It has been reported that NSN1 in tobacco (*Nicotiana tabacum*) and Arabidopsis plays an important role in 27S rRNA precursor processing and 60S large subunit assembly (Jeon et al., 2015). Its human ortholog GNL3L can associate with early pre‐60S particles and participates in 60S subunit assembly (Thomé et al., 2026), while its yeast ortholog Nug1p specifically binds to late pre‐60S particles and is primarily involved in 60S subunit assembly and nuclear export (Bassler et al., 2001). Notably, yeast Nug1p was also identified as a candidate interactor of Npa1p, and the rRNA processing defects of *nug1* and *npa1* mutants are nearly identical (Bassler et al., 2001; Dez et al., 2004; Manikas et al., 2016). Furthermore, Nug1p regulates the stable association of the helicase Dbp10 with pre‐60S particles through its nucleotide‐binding ability, thereby participating in peptidyl transferase center maturation (Manikas et al., 2016). Similarly, if Npa1L1 indeed participates in 27S rRNA precursor processing and 60S subunit assembly, it may recruit NSN1 and stabilize the positioning of RNA helicases on 60S particles to coordinately facilitate this process.

Taken together, RH7 may primarily participate in 18S rRNA precursor processing and 40S subunit assembly; RH27 may be involved in both 18S and 27S rRNA precursor processing as well as 40S and 60S subunit assembly; and NSN1 is likely mainly engaged in 27S rRNA precursor processing and 60S subunit assembly and export. Based on the two possible functions of Arabidopsis Npa1L1 in rRNA processing and ribosomal subunit assembly, we propose two possible models. In the first, Npa1L1 acts as a scaffold to recruit the DEAD‐box RNA helicases RH7 and RH27, utilizing their helicase activities to promote 18S rRNA precursor processing and 40S subunit assembly. In the second, Npa1L1 recruits NSN1 and stabilizes the positioning of RH27 on 60S particles, jointly participating in 27S rRNA precursor processing and 60S subunit assembly and export. However, which of these models reflects the actual mechanism, and how Npa1L1 coordinates with RH7, RH27, and NSN1 to execute their functions, remains to be further elucidated through future studies using inducible knockdown systems and RNA–protein binding assays to define the precise functions of Npa1L1, RH7, and RH27, which also constitutes a limitation of the present work.

Mutation of Arabidopsis Npa1L1 leads to pronounced accumulation of 35S and P‐A_3_ pre‐rRNAs. Notably, this phenotype has also been observed in mutants of several other Arabidopsis ribosome biogenesis factors, including RRP7 (Ribosomal RNA Processing 7), IRP5 (Involved in rRNA Processing 5), and NOB1 (Nin1 One Binding Protein 1) (Micol‐Ponce et al., 2018; Missbach et al., 2013; Palm et al., 2019). Future work will explore whether these proteins also interact with Npa1L1 to jointly modulate rRNA processing.

### Ribosome biogenesis factor Npa1L1 affects embryo development

Many essential ribosome biogenesis factors in Arabidopsis are indispensable for plant growth and embryo development (Byrne, 2009; Dörner et al., 2023; Palm et al., 2019; Weis et al., 2015). We found that Npa1L1 mutation reduces the abundance of 80S ribosomal monomers compared to WT, which may compromise overall protein synthesis efficiency.

Early embryos undergo vigorous cell division and require massive protein production; in particular, the globular embryo stage is critical for rapid cell division, pattern formation, and organ primordia establishment (ten Hove et al., 2015; Tian et al., 2020). Accordingly, the delayed cell division and asymmetric development observed in *npa1l1* mutant embryos starting from the globular stage (Figure 2a–u) are likely attributable to insufficient synthesis of proteins essential for embryogenesis.

In summary, this study demonstrates that the protein encoded by the previously uncharacterized gene *EMB2788* (*Npa1L1*) in Arabidopsis acts as a crucial ribosome biogenesis factor involved in pre‐rRNA processing. It may collaborate with RH7, RH27, and NSN1 to modulate pre‐rRNA processing and participate in ribosomal subunit assembly. This work identifies a novel key factor involved in plant ribosome biogenesis, refines the regulatory network of pre‐rRNA processing in plants, and provides a theoretical basis for further elucidating the molecular mechanisms underlying plant ribosome biogenesis.

## MATERIALS AND METHODS

### Plant materials and growth conditions

All Arabidopsis plants used in this study were of the Columbia (Col‐0) ecotype. The T‐DNA insertion mutants *npa1l1–1* (SALK_007803), *npa1l1–2* (SALK_021117), *rh7‐3* (SALK_018195), *rh27‐2* (SALK_068661), and *nsn1‐1* (SALK_029201) were obtained from the Arabidopsis Biological Resource Center (ABRC, https://www.Arabidopsis.org).

Seeds were surface‐sterilized with 75% ethanol, rinsed three times with sterile water, and sown on MS medium (4.33 g/L Murashige and Skoog basal salt mixture, 0.75% agar, 2% sucrose, pH 5.75). Plates were kept at 4°C for 3 days and then transferred to growth chambers under long‐day conditions (16 h light/8 h dark) at 22°C. After 10 days of growth on MS medium, seedlings were transplanted into soil and grown in a greenhouse (22°C, long‐day conditions). Transgenic seedlings expressing p35S::GFP‐Npa1L1, p35S::RH7‐MYC, p35S::RH27‐MYC, p35S::NSN1‐MYC, and p35S::GFP were grown for 10 days on MS medium and collected for co‐immunoprecipitation assays. Mutant seedlings (*npa1l1‐1*, *npa1l1‐2*, *rh7‐3*, *rh27‐2*, and *nsn1‐1*) were grown for 20 days on MS medium and harvested for cRT‐PCR or northern blot analysis.

### Genotyping analysis

Genomic DNA was extracted from leaves as previously described (Edwards et al., 1991). T‐DNA insertions were analyzed by PCR genotyping using gene‐specific primers listed in Table S1.

### Molecular cloning

To create pNpa1L1::GFP‐Npa1L1, a promoter fragment containing 2001 bp nucleotides (upstream of the *Npa1L1* start codon) was amplified by PCR and cloned using PstI/XbaI into the pCambia1300‐Pro35S::GFP binary vector to generate pNpa1L1::GFP. Then, Npa1L1 genomic DNA was amplified and inserted into the pNpa1L1::GFP vector using BamHI/SmaI to generate pNpa1L1::GFP‐Npa1L1.

To create p35S::GFP‐Npa1L1, Npa1L1 genomic DNA was amplified and cloned into the pCambia1300‐Pro35S::GFP binary vector using BamHI/SmaI. To create p35S::RH7‐MYC, p35S::RH27‐MYC, and p35S::NSN1‐MYC, the CDS sequences of *RH7* (2013 bp), *RH27* (1899 bp), and *NSN1* (1746 bp) were amplified and cloned into the pCambia1300‐Pro35S::MYC binary vector using PstI/BamHI, PstI/XbaI, and PstI/XbaI, respectively.

### Plant transformation and selection

All constructs were introduced into Arabidopsis via *Agrobacterium tumefaciens* (strain GV3101)‐mediated floral dip transformation (Clough & Bent, 1998). Transformants were selected on MS medium containing 25 mg·L^−1^ hygromycin B (Calbiochem, San Diego, CA, USA).

### Observation of embryo development

To track embryo development, open flowers from WT and mutant plants were marked daily with colored threads at 10 AM for 8 days. Marked siliques were harvested, and carpels were carefully dissected open under a stereomicroscope using a dissecting needle. Siliques were fixed in a solution of 4% glutaraldehyde and 0.1% Triton X‐100 in PBS (pH 7.4) under vacuum overnight. Samples were then cleared in a chloral hydrate:water:glycerol solution (8:2:1, w/v/v) for several minutes to hours, depending on the embryo stage. Ovules containing embryos were dissected from siliques and imaged using differential interference contrast (DIC) microscopy (Zeiss AXIO Imager A1).

### Subcellular localization analysis

The pNpa1L1::GFP‐Npa1L1 construct was transformed into *npa1l1/+* plants. GFP signals in T3 transgenic plants were observed by CLSM (GFP: excitation 488 nm, emission 505–530 nm; DAPI: excitation 405 nm, emission 410–585 nm) using a FV3000 confocal laser scanning microscope (CLSM; Olympus).

### Polysome profiling

Polysomes were fractionated over sucrose gradients as previously described with minor modifications (Chassé et al., 2017; Mustroph et al., 2009; Poria & Ray, 2017). A total of 5000 A260 units of the supernatant were layered onto a linear 10%–45% sucrose gradient poured with the Gradient Master 108 (BioComp Instruments), then ultracentrifuged in a Beckman SW‐41Ti rotor at 36000 rpm for 3 h at 4°C. The gradients were analyzed using the Piston Gradient Fractionator (BioComp Instruments) attached to a Model EM‐1 Econo UV Monitor (Bio‐Rad) for continuous measurement of the absorbance at 260 nm.

### cRT‐PCR

Total RNA was extracted using an Eastep Super Total RNA Extraction Kit (LS1040; Promega) according to the manufacturer's instructions. cRT‐PCR was performed as described previously (Hang et al., 2015).

### Northern blot

Northern blot analyses were conducted using digoxigenin (DIG)‐labeled oligonucleotide probes S1 and S2 (3′‐DIG‐labeled, synthesized by Sangon Biotech, Shanghai, China). Total RNA was extracted using an Eastep Super Total RNA Extraction Kit (LS1040; Promega). Ten micrograms of total RNA was separated on a 1.5% (wt/vol) agarose/formaldehyde gel and transferred via capillary elution to a nylon membrane (Thermo Fisher Scientific). The membrane was prehybridized for 2 h at 65°C and hybridized overnight at 65°C with 25 ng·mL^−1^ of probe. The DIG luminescent signals were detected by the DIG Luminescent Detection Kit according to the manufacturer's instructions (Cat. No. 11363514910, Roche).

### Co‐IP assay

The p35S::GFP‐Npa1L1, p35S::RH7‐MYC, p35S::RH27‐MYC, and p35S::NSN1‐MYC plasmids were transformed into WT plants, respectively. Plants expressing p35S::GFP‐Npa1L1 were crossed with plants expressing p35S::RH7‐MYC, p35S::RH27‐MYC, and p35S::NSN1‐MYC, respectively, to obtain co‐expression plants. The nuclear proteins were extracted from co‐expression seedlings and were pre‐incubated with 10 μg/mL RNase A at 4°C for 1 h. GFP‐Npa1L1 protein was immunopurified using GFP‐Trap magnetic agarose beads according to the manufacturer's instructions (gtma, ChromoTek). The eluate of GFP‐Npa1L1 protein was divided into two parts and both were separated on SDS‐polyacrylamide gel electrophoresis (PAGE) gels which were transferred to two polyvinylidene difluoride (PVDF) membranes (0.45 μm, Millipore). One membrane was hybridized with the primary anti‐GFP antibody (AG5001, TongBio Pacific) followed by treatment with the secondary goat anti‐rabbit IgG antibody (33101ES60, Yeasen). The other membrane was hybridized with the primary anti‐MYC antibody (M192‐3, MBL) followed by treatment with the secondary goat anti‐mouse IgG antibody (ZB2305, Zhongshan Jinqiao Biological Technology). The band signals were detected with a Super Signal™ West Femto Trial Kit (34 096, Thermo).

### Primers, probes, and constructs

Primers used for genotyping T‐DNA mutants and cRT‐PCR, as well as probes for northern blot, are listed in Table S1. Primers used for plasmid construction are provided in Table S2.

### Accession numbers

The accession numbers of genes referred in this work were as follows: *Npa1L1*, AT4G27010; *RH7*, At5G62190; *RH27*, AT5G65900; *NSN1*, At3G07050.

## Author Contributions

RL, YG, and ML conceived the project and designed the experiments. ML, ZJL, and NC carried out the phenotypic analysis and embryo observation. ML, SLW, and ZXL carried out the northern blot, cRT‐PCR, and Co‐IP assays. MJT and AWZ carried out the embryo fluorescence detection assays. YRZ and JSZ carried out the complementation tests and subcellular localization assays. LFC carried out the forward genetic screen assay. RL, YG, and ML wrote the manuscript.

## Conflict of Interest

The authors declare no conflict of interest.

## Supporting information


**Figure S1.** Domain analysis and amino acid sequence alignment of the Npa1 and NopRA1 domains. (a) The Npa1 and NopRA1 domains of Npa1 proteins in *Arabidopsis thaliana* Npa1L1, *Arabidopsis thaliana* Npa1L2, *Glycine max*, *Sorghum bicolor*, *Danio rerio* (Zebrafish), *Homo sapiens*, and *Saccharomyces cerevisiae*. The Npa1 domain is indicated with green frames and the NopRA1 domain is marked with blue frames, which were predicted by InterPro (https://www.ebi.ac.uk/interpro/). The protein molecular weight is labeled. (b, c) Amino acid sequence alignments of the Npa1 and NopRA1 domains in *Arabidopsis thaliana* Npa1L1, *Arabidopsis thaliana* Npa1L2, *Glycine max*, *Sorghum bicolor*, *Danio rerio* (Zebrafish), *Homo sapiens*, and *Saccharomyces cerevisiae*. Black frames indicate amino acids that are completely identical across different species. Pink frames indicate amino acids with a conservation rate of ≥75% among different species. Blue frames indicate amino acids with a conservation rate of ≥50% among different species.
**Figure S2.** Phylogenetic analysis of Npa1L1 and its homologs. Phylogenetic relationships of Npa1L1 and homologous proteins across plants, animals, and fungi. The neighbor‐joining phylogenetic tree was constructed using MEGA 6.06, based on protein sequences from *Arabidopsis thaliana*, *Glycine max*, *Sorghum bicolor*, *Zostera marina*, *Gallus gallus*, *Danio rerio* (Zebrafish), *Homo sapiens*, and *Saccharomyces cerevisiae*. The Npa1L1 protein is marked with a green solid triangle.
**Figure S3.** Domain analysis and amino acid sequence alignment of Arabidopsis Npa1L1/Npa1L2 and *Saccharomyces cerevisiae* Npa1p. (a) Amino acid sequence alignment of Arabidopsis Npa1L1 and Npa1L2 proteins. The two paralogs share 66.01% amino acid identity. The Npa1 domains of the two paralogs share 69.93% amino acid sequence identity. The NopRA1 domains of the two paralogs share 71.23% amino acid sequence identity. Conserved residues are boxed in black. Red lines indicate the positions of the domains. (b, c) Amino acid sequence alignments of the Npa1 and NopRA1 domains from Arabidopsis Npa1L1 and *Saccharomyces cerevisiae* Npa1p, respectively. The Npa1 domains show 17.72% sequence identity, whereas the NopRA1 domains show 15.88% sequence identity. Conserved residues are boxed in black.
**Figure S4.** Expression profile of *Npa1L1* and *Npa1L2* (Winter et al., 2007) The expression profile of *Npa1L1* (a) and *Npa1L2* (b) was obtained from Arabidopsis eFP Browser (https://bar.utoronto.ca/eplant/).
**Figure S5.** Genotyping analysis of *npa1l1–1*, *npa1l1–2*, and functionally complemented lines. (a) Schematic diagram of the Npa1L1 genomic sequence structure and each T‐DNA insertion site. Primers *npa1l1‐1*‐FP/*npa1l1‐1*‐RP and *npa1l1‐2*‐FP/*npa1l1‐2*‐RP were designed for the genotyping analysis of *npa1l1‐1* and *npa1l1‐2* mutants, respectively. Primers *npa1l1‐Comp*‐FP/*npa1l1‐Comp*‐RP were designed for the genotyping analysis of functionally complemented (Comp) *npa1l1* transgenic plants. Black boxes represent exons, gray lines represent introns, and the white box represents the untranslated region (UTR). (b) PCR analysis of *npa1l1‐1* and *npa1l1‐2* mutants. (c) PCR analysis of seedlings with abnormal germination. (d) PCR analysis of pNpa1L1::GFP‐Npa1L1 functionally complemented lines (Comp) in the *npa1l1‐1* and *npa1l1‐2* backgrounds.
**Figure S6.** The embryo development of *npa1l1/+* mutants was normal from the zygote to the 2/4‐cell stage. (a, d, g) The embryos of WT, *npa1l1–1/+*, and *npa1l1–2/+* at the zygote stage, respectively. Scale bar = 10 μm. (b, e, h) The embryos of WT, *npa1l1–1/+*, and *npa1l1–2/+* at the 1‐cell stage, respectively. Scale bar = 10 μm. (c, f, i) The embryos of WT, *npa1l1–1/+*, and *npa1l1–2/+* at the 2/4‐cell stage, respectively. Scale bar = 10 μm.
**Figure S7.** Endosperm development was perturbed in *npa1l1/+* embryos. (a, e, i) Endosperm of WT, *npa1l1–1/+*, and *npa1l1–2/+* plants at the zygote. White asterisks indicate endosperm nuclei. White frames mark magnified views shown as (b, f, j), respectively. Scale bars in (a, e, i) = 20 μm. Scale bars in (b, f, j) = 10 μm. (c, g, k) Endosperm of WT, *npa1l1–1/+*, and *npa1l1–2/+* plants at the late globular. White frames mark magnified views shown as (d, h, l), respectively. Scale bars in (c, g, k) = 50 μm. Scale bars in (d, h, l) = 20 μm. (m, q, u) Endosperm of WT, *npa1l1–1/+*, and *npa1l1–2/+* plants at the heart. White frames mark magnified views shown as (n, r, v), respectively. Scale bars in (m, q, u) = 50 μm. Scale bars in (n, r, v) = 20 μm. (o, s, w) Endosperm of WT, *npa1l1–1/+*, and *npa1l1–2/+* plants at the heart. White frames mark magnified views shown as (p, t, x), respectively. Scale bars in (o, s, w) = 50 μm. Scale bars in (p, t, x) = 20 μm.
**Figure S8.** Proposed roles of Arabidopsis Npa1L1 in pre‐rRNA processing based on the molecular phenotype of *npa1l1* mutants. Northern blot and circular RT‐PCR analyses revealed pre‐rRNA species that are accumulated or reduced (red arrows) in the *npa1l1* mutant relative to WT. Single and double red arrows denote mild and significant increases in pre‑rRNA abundance in the *npa1l1* mutant, respectively. Arabidopsis Npa1L1 mainly affects 35S and 18S (P‐A_3_) pre‐rRNAs processing, as both precursors accumulate in the mutants (detected by r1/r2 and r5/r6 primers and probes S1–S3). Given its potential role in P′ cleavage in the ITS1‐first pathway, Npa1L1 may also cleave the same site in the 5′ETS‐first pathway.
**Figure S9.** Summary of the roles of yeast Npa1p in pre‐rRNA processing (Dez et al., 2004; Rosado & De La Cruz, 2004; Choque et al., 2018; An et al., 2024). Loss of yeast Npa1p function results in aberrant accumulation or altered processing of several rRNA precursors, including 35S pre‐rRNA, 27SA_2_ pre‐rRNA, and the aberrant 23S intermediate. In *npa1p* mutants, the levels of 27SA_2_ intermediates are decreased, which is caused by abnormal cleavage at the A_2_ site. Meanwhile, the 35S pre‐rRNA and 18S rRNA precursors (23S) are overaccumulated and 18S rRNA was reduced, resulting from abnormal cleavage at the A_0_, A_3_, and D sites, respectively. Red arrows indicate the pre‐rRNAs that are overaccumulated or depleted in *npa1p* mutants (Dez et al., 2004). Blue arrows indicate the pre‐rRNAs that are overaccumulated or depleted in *npa1p* mutants (Rosado & De La Cruz, 2004). Single red or blue arrow indicates mild alteration in pre‐rRNAs abundance, while double arrows denote significant change. The sites in parentheses correspond to the corresponding sites in Arabidopsis.
**Table S1.** Primers used for identification of T‐DNA insertion mutants and amplification of cRT‐PCR, and probes sequences for hybridization of northern blot.
**Table S2.** Primers used for constructs.

## Data Availability

All relevant data can be found within the manuscript and its supporting materials. The materials that support the findings of this study are available from the corresponding author upon request.
