# Supplementary material for: Arabidopsis EMB2788/Npa1L1 is critical for pre‐rRNA processing, ribosome biogenesis, and embryo development
Source: Plant J. 2026 Sep 9;127(5):e71114. doi: 10.1111/tpj.71114 (PMC13559043; doi:10.1111/tpj.71114)
Supplement: Supplementary file 1 [file TPJ-127-0-s001.pdf]

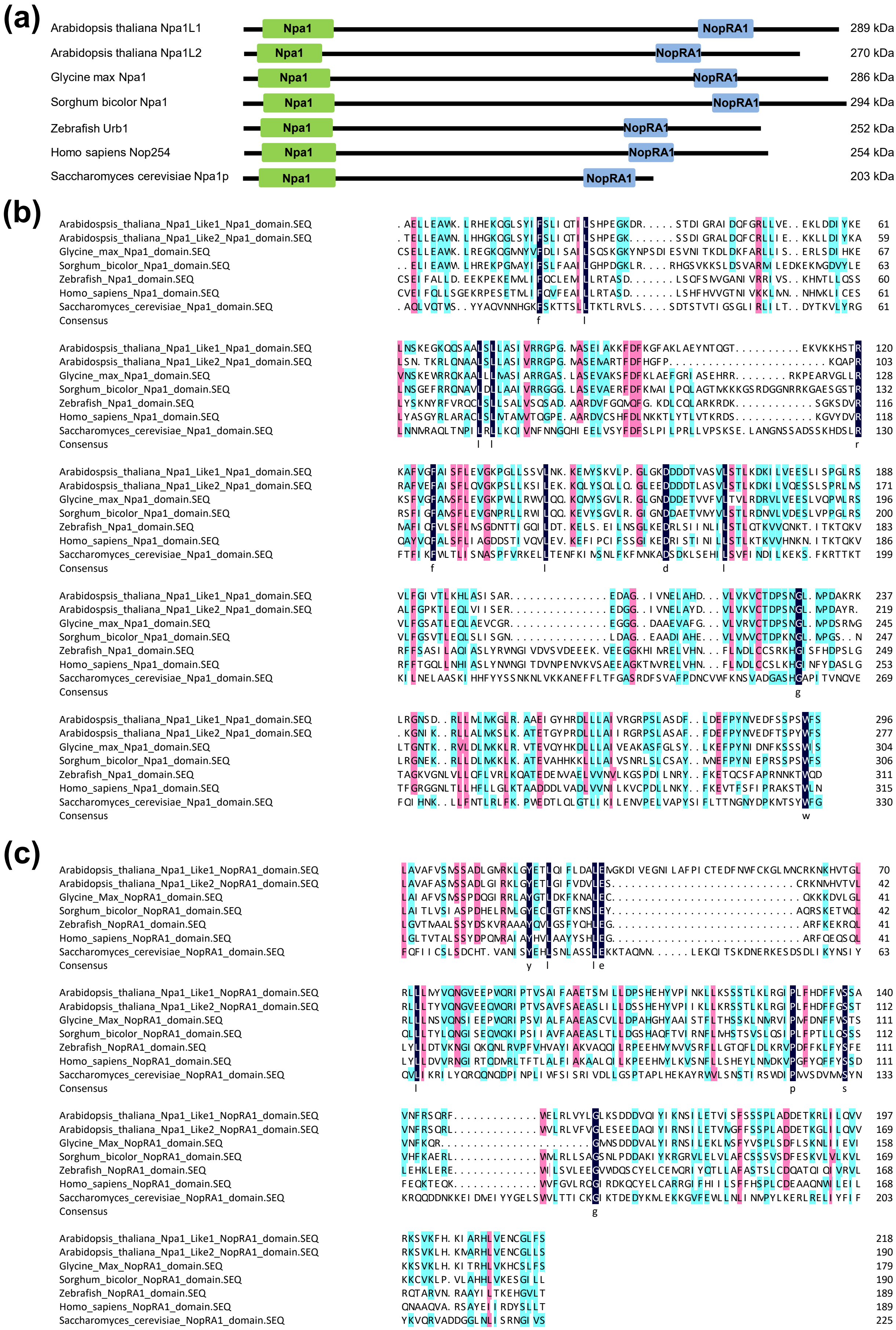

(a)
Arabidopsis thaliana Npa1L1
Arabidopsis thaliana Npa1L2
Glycine max Npa1
Sorghum bicolor Npa1
Zebrafish Urb1
Homo sapiens Nop254
Saccharomyces cerevisiae Npa1p
Npa1
NopRA1
289 kDa
270 kDa
286 kDa
294 kDa
252 kDa
254 kDa
203 kDa
(b)
Arabidospsis_thaliana_Npa1_Like1_Npa1_domain.SEQ
Arabidospsis_thaliana_Npa1_Like2_Npa1_domain.SEQ
Glycine_max_Npa1_domain.SEQ
Sorghum_bicolor_Npa1_domain.SEQ
Zebrafish_Npa1_domain.SEQ
Homo_sapiens_Npa1_domain.SEQ
Saccharomyces_cerevisiae_Npa1_domain.SEQ
Consensus
(c)
Arabidospsis_thaliana_Npa1_Like1_NopRA1_domain.SEQ
Arabidospsis_thaliana_Npa1_Like2_NopRA1_domain.SEQ
Glycine_Max_NopRA1_domain.SEQ
Sorghum_bicolor_NopRA1_domain.SEQ
Zebrafish_NopRA1_domain.SEQ
Homo_sapiens_NopRA1_domain.SEQ
Saccharomyces_cerevisiae_NopRA1_domain.SEQ
Consensus

Figure S1. Domain analysis and amino acid sequence alignment of the Npa1 and NopRA1 domains.

(a) The Npa1 and NopRA1 domains of Npa1 proteins in *Arabidopsis thaliana* Npa1L1, *Arabidopsis thaliana* Npa1L2, *Glycine max*, *Sorghum bicolor*, *Danio rerio* (Zebrafish), *Homo sapiens*, and *Saccharomyces cerevisiae.* The Npa1 domain is indicated with green frames and the NopRA1 domain is marked with blue frames, which were predicted by InterPro (http://www.ebi.ac.uk/interpro/). The protein molecular weight is labeled.
(b, c) Amino acid sequence alignments of the Npa1 and NopRA1 domains in *Arabidopsis thaliana* Npa1L1, *Arabidopsis thaliana* Npa1L2, *Glycine max*, *Sorghum bicolor*, *Danio rerio* (Zebrafish), *Homo sapiens*, and *Saccharomyces cerevisiae*. Black frames indicate amino acids that are completely identical across different species. Pink frames indicate amino acids with a conservation rate of ≥ 75% among different species. Blue frames indicate amino acids with a conservation rate of ≥ 50% among different species.

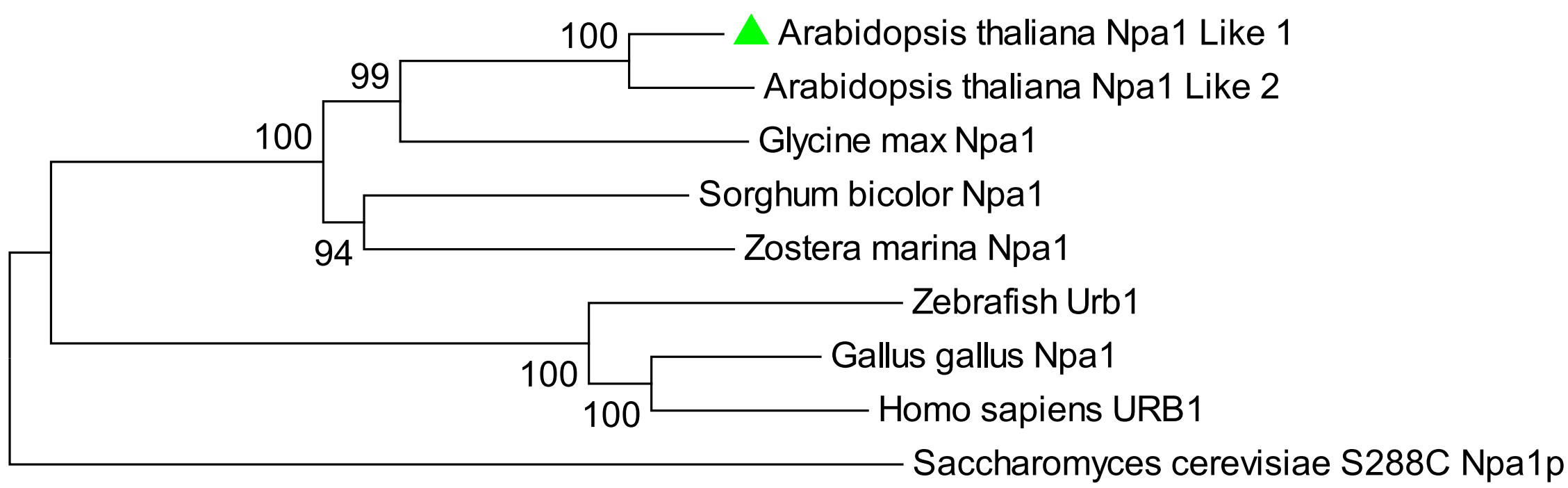

Figure S2. Phylogenetic analysis of Npa1L1 and its homologs.

Phylogenetic relationships of Npa1L1 and homologous proteins across plants, animals, and fungi. The neighbor-joining phylogenetic tree was constructed using MEGA 6.06, based on protein sequences from *Arabidopsis thaliana*, *Glycine max*, *Sorghum bicolor*, *Zostera marina*, *Gallus gallus*, *Danio rerio* (Zebrafish), *Homo sapiens*, and *Saccharomyces cerevisiae*. The Npa1L1 protein is marked with a green solid triangle.

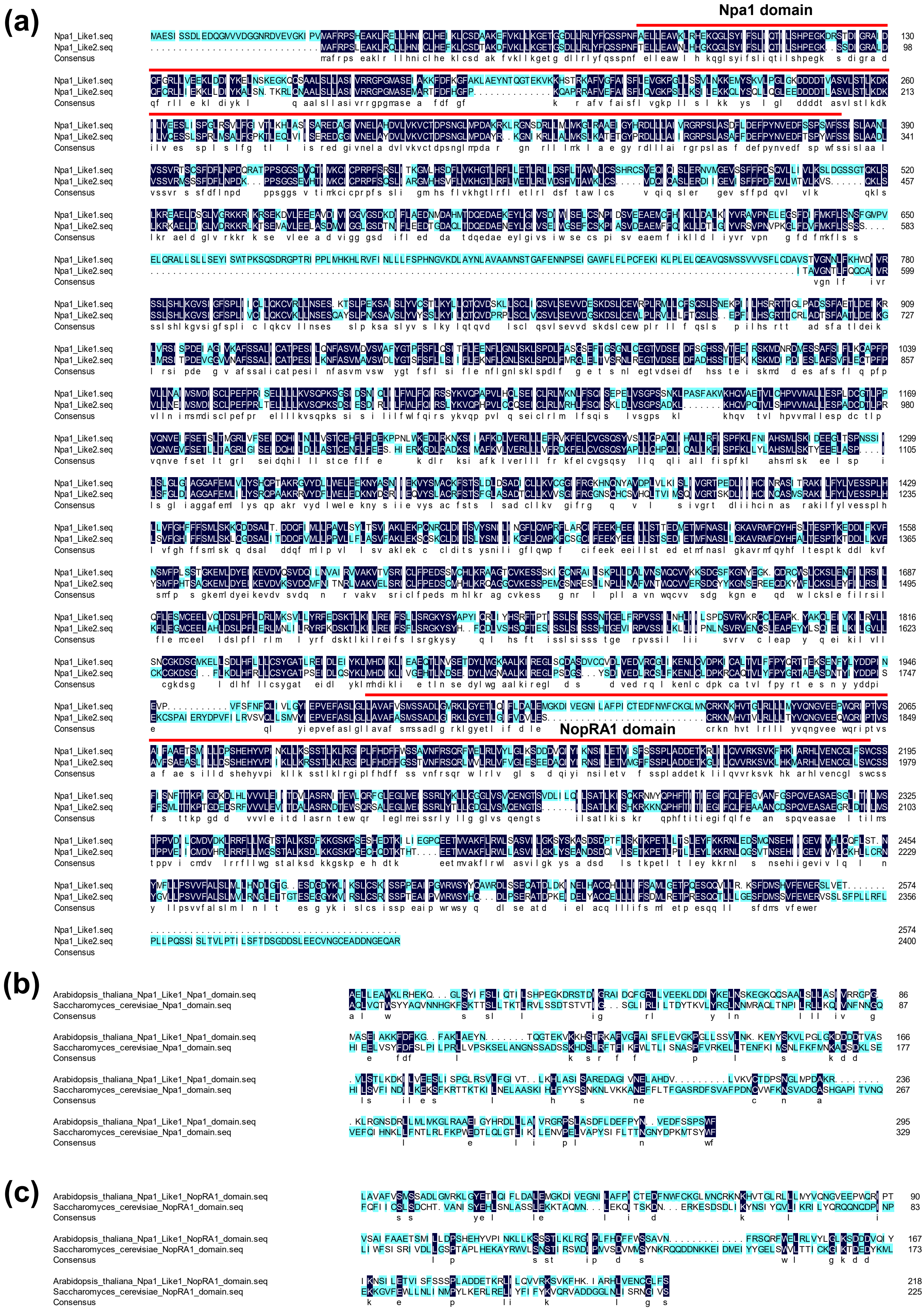

(a)
Npa1 domain
Npa1_Like1.seq
Npa1_Like2.seq
Consensus
NopRA1 domain
(b)
Arabidopsis_thaliana_Npa1_Like1_Npa1_domain.seq
Saccharomyces_cerevisiae_Npa1_domain.seq
Consensus
(c)
Arabidopsis_thaliana_Npa1_Like1_NopRA1_domain.seq
Saccharomyces_cerevisiae_NopRA1_domain.seq
Consensus

Figure S3. Domain analysis and amino acid sequence alignment of Arabidopsis Npa1L1/Npa1L2 and *Saccharomyces cerevisiae* Npa1p.

(a) Amino acid sequence alignment of Arabidopsis Npa1L1 and Npa1L2 proteins. The two paralogs share 66.01% amino acid identity. The Npa1 domains of the two paralogs share 69.93% amino acid sequence identity. The NopRA1 domains of the two paralogs share 71.23% amino acid sequence identity. Conserved residues are boxed in black. Red lines indicate the positions of the domains.
(b, c) Amino acid sequence alignments of the Npa1 and NopRA1 domains from Arabidopsis Npa1L1 and *Saccharomyces cerevisiae* Npa1p, respectively. The Npa1 domains show 17.72% sequence identity, whereas the NopRA1 domains show 15.88% sequence identity. Conserved residues are boxed in black.

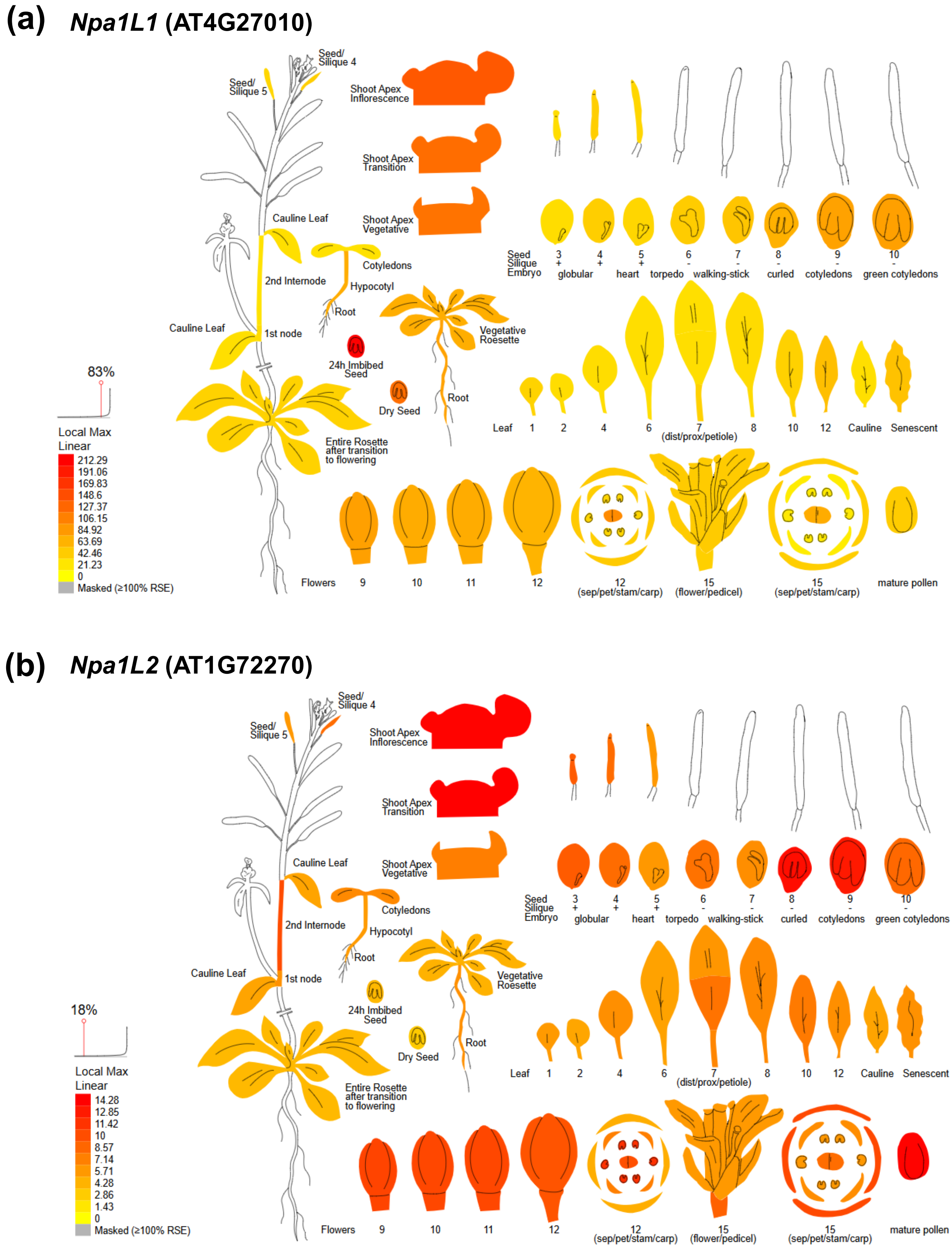

Figure S4. Expression profile of *Npa1L1* and *Npa1L2* (Winter et al., 2007)

The expression profile of *Npa1L1* **(a)** and *Npa1L2* **(b)** was obtained from Arabidopsis eFP Browser (http://bar.utoronto.ca/eplant/).

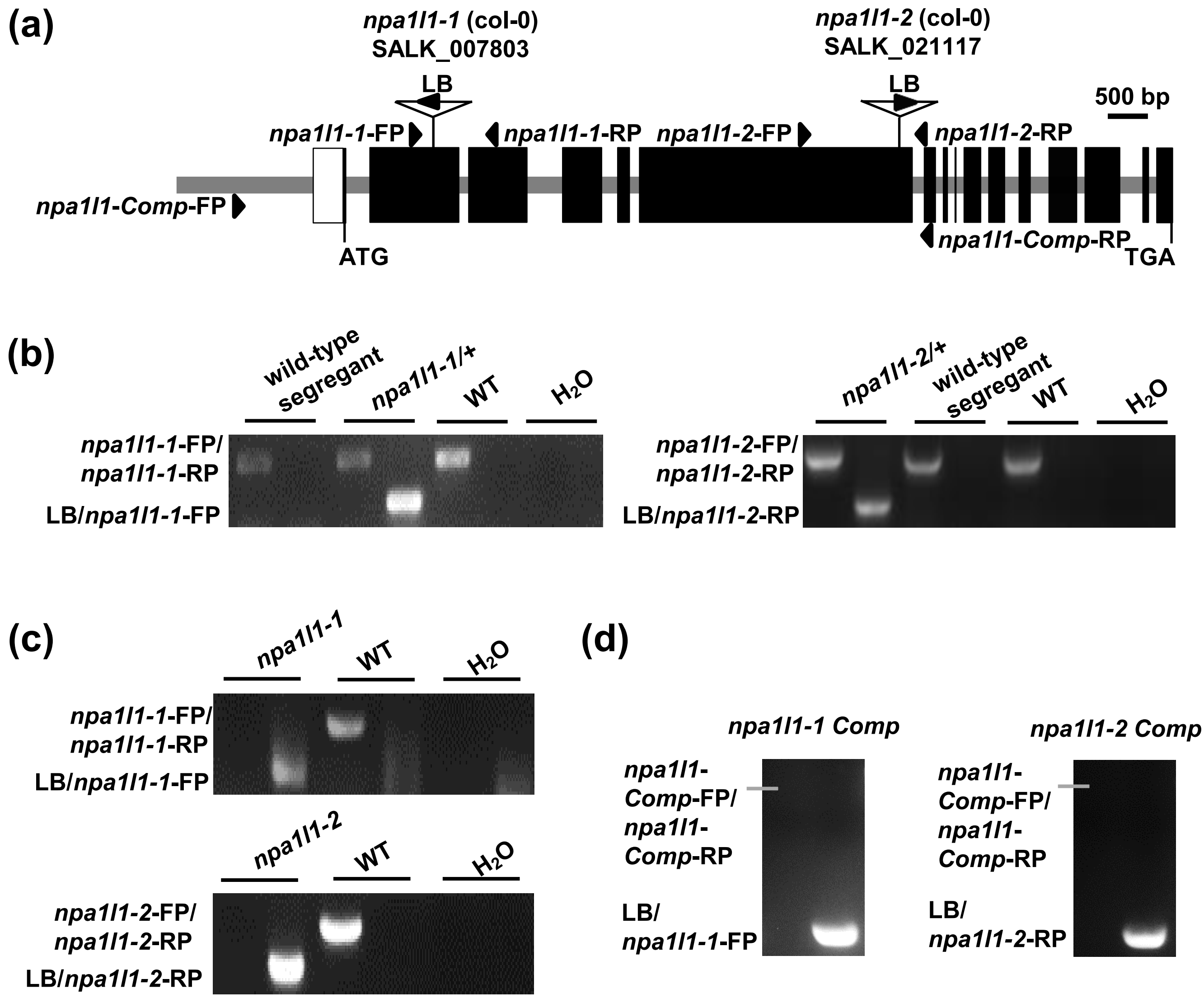

Figure S5. Genotyping analysis of *npa1l1-1*, *npa1l1-2*, and functionally complemented lines.

(a) Schematic diagram of the Npa1L1 genomic sequence structure and each T-DNA insertion site. Primers *npa1l1-1*-FP/*npa1l1-1*-RP and *npa1l1-2*-FP/*npa1l1-2*-RP were designed for the genotyping analysis of *npa1l1-1* and *npa1l1-2* mutants, respectively. Primers *npa1l1-Comp*-FP/*npa1l1-Comp*-RP were designed for the genotyping analysis of functionally complemented (Comp) *npa1l1* transgenic plants. Black boxes represent exons, gray lines represent introns, and the white box represents the untranslated region (UTR).

(b) PCR analysis of *npa1l1-1* and *npa1l1-2* mutants.

(c) PCR analysis of seedlings with abnormal germination.

(d) PCR analysis of pNpa1L1::GFP-Npa1L1 functionally complemented lines (Comp) in the *npa1l1-1* and *npa1l1-2* backgrounds.

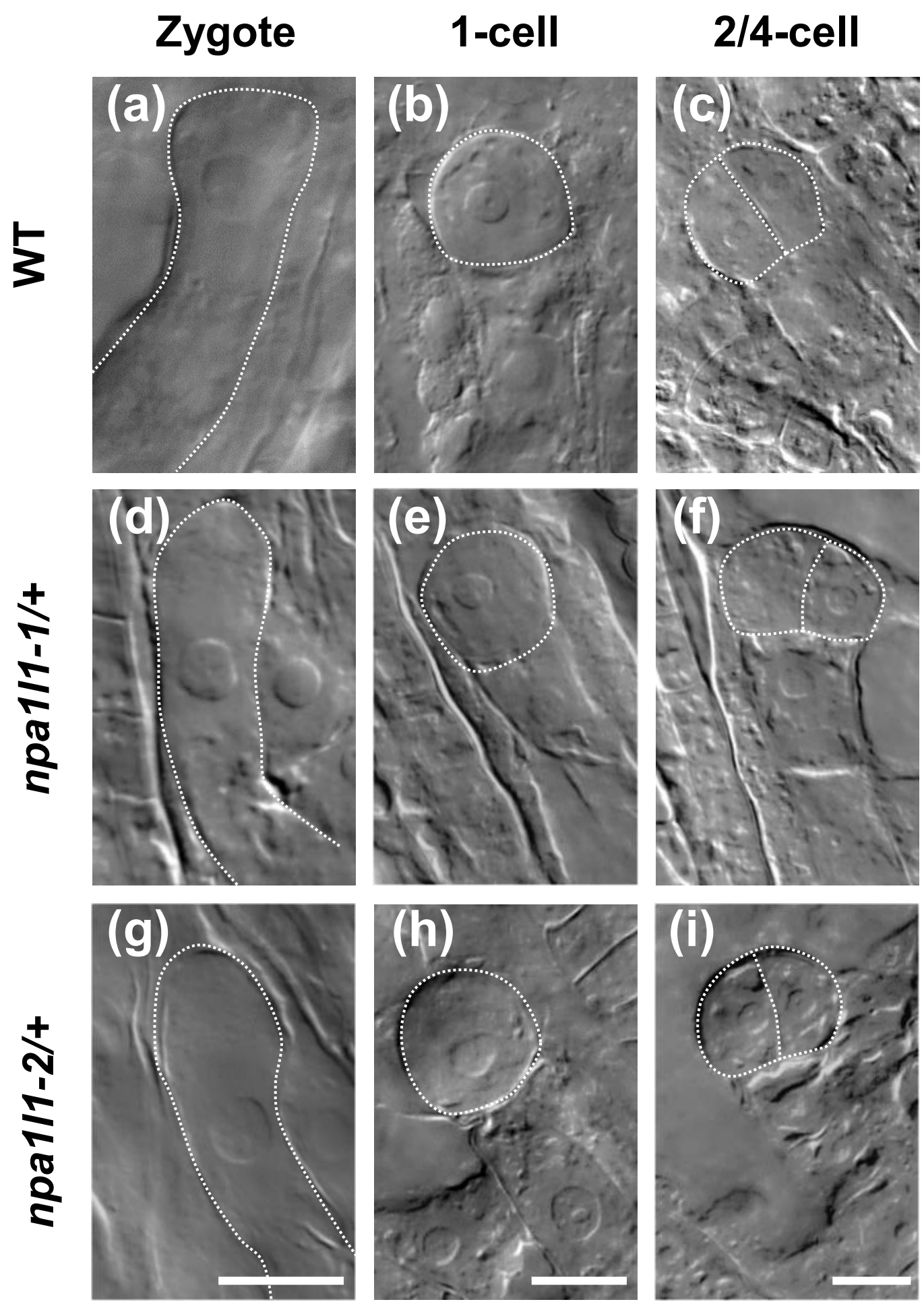

Figure S6. The embryo development of *npa1l1/+* mutants was normal from the zygote to the 2/4-cell stage.

(a, d, g) show the embryos of WT, *npa1l1-1/*+, and *npa1l1-2/*+ at the zygote stage, respectively. Scale bar = 10 μm.
(b, e, h) show the embryos of WT, *npa1l1-1/*+, and *npa1l1-2/*+ at the 1-cell stage, respectively. Scale bar = 10 μm.
(c, f, i) show the embryos of WT, *npa1l1-1/*+, and *npa1l1-2/*+ at the 2/4-cell stage, respectively. Scale bar = 10 μm.

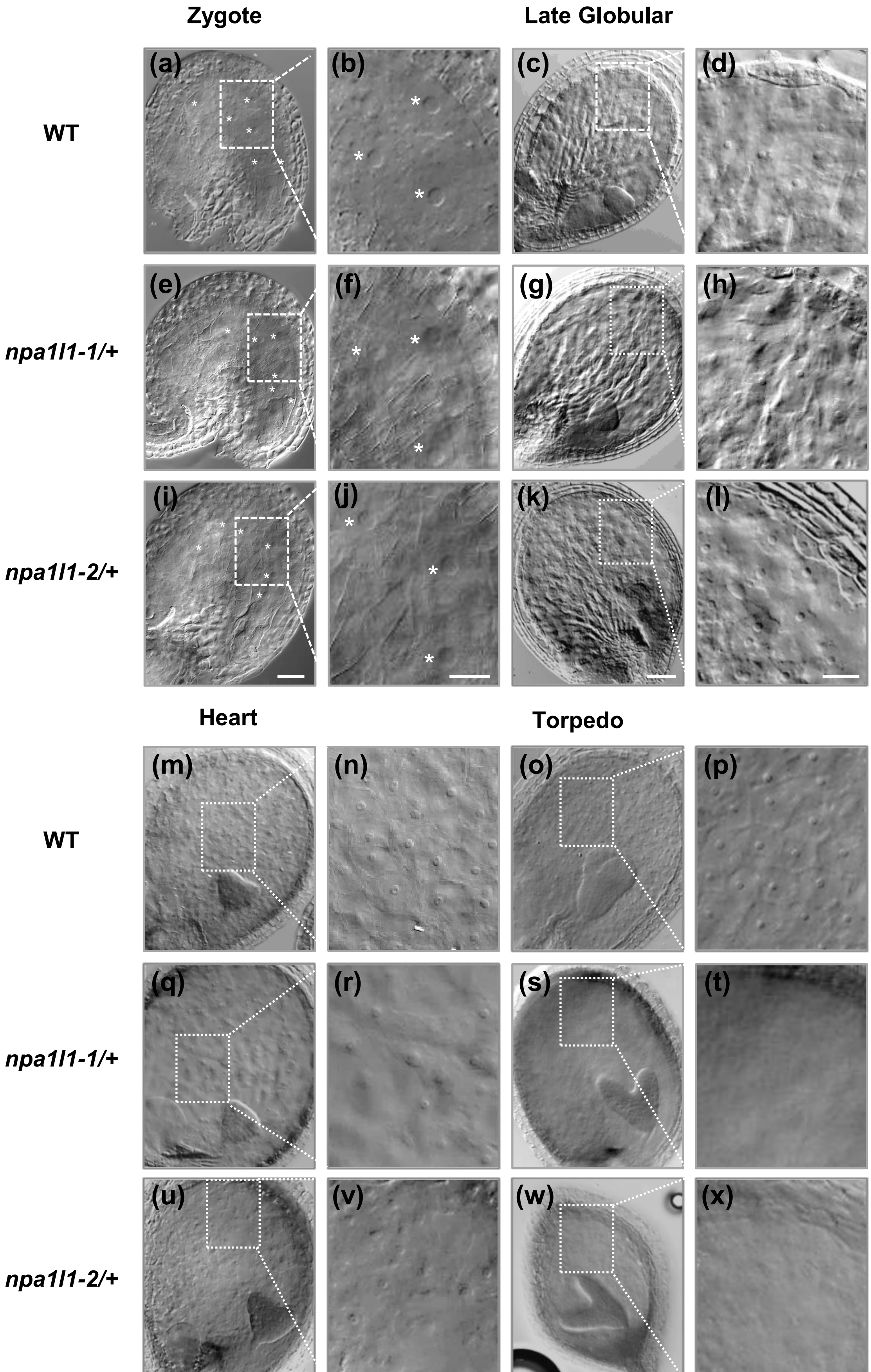
Zygote
Late Globular
WT
npa1l1-1/+
npa1l1-2/+
(a)
(b)
(c)
(d)
(e)
(f)
(g)
(h)
(i)
(j)
(k)
(l)
Heart
Torpedo
WT
npa1l1-1/+
npa1l1-2/+
(m)
(n)
(o)
(p)
(q)
(r)
(s)
(t)
(u)
(v)
(w)
(x)

Figure S7. Endosperm development was perturbed in *npa1l1/+* embryos*.*

(a, e, i) Endosperm of WT, *npa1l1-1/+*, and *npa1l1-2/+* plants at the zygote. White asterisks indicate endosperm nuclei. White frames mark magnified views shown as **(b, f, j)**, respectively. Scale bars in **(a, e, i)** = 20 μm. Scale bars in **(b, f, j)** = 10 μm.
(c, g, k) Endosperm of WT, *npa1l1-1/+*, and *npa1l1-2/+* plants at the late globular. White frames mark magnified views shown as **(d, h, l)**, respectively. Scale bars in **(c, g, k)** = 50 μm. Scale bars in **(d, h, l)** = 20 μm.
(m, q, u) Endosperm of WT, *npa1l1-1/+*, and *npa1l1-2/+* plants at the heart. White frames mark magnified views shown as **(n, r, v)**, respectively. Scale bars in **(m, q, u)** = 50 μm. Scale bars in **(n, r, v)** = 20 μm.
(o, s, w) Endosperm of WT, *npa1l1-1/+*, and *npa1l1-2/+* plants at the heart. White frames mark magnified views shown as **(p, t, x)**, respectively. Scale bars in **(o, s, w)** = 50 μm. Scale bars in **(p, t, x)** = 20 μm.

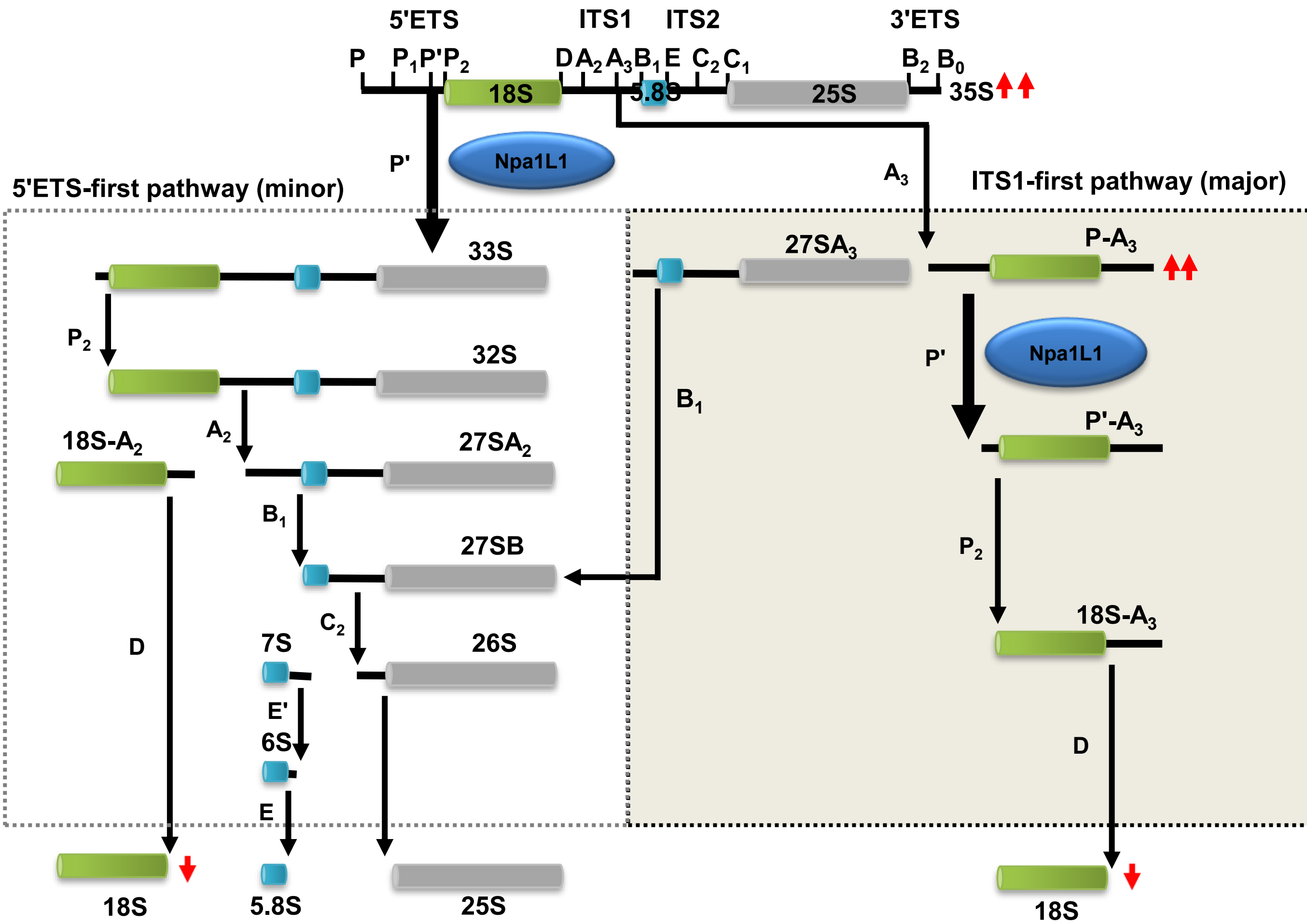

Figure S8. Proposed roles of Arabidopsis Npa1L1 in pre-rRNA processing based on the molecular phenotype of *npa1l1* mutants.

Northern blot and circular RT-PCR analyses revealed pre-rRNA species that are accumulated or reduced (red arrows) in the *npa1l1* mutant relative to WT. Single and double red arrows denote mild and significant increases in pre-rRNA abundance in the *npa1l1* mutant, respectively. Arabidopsis Npa1L1 mainly affects 35S and 18S (P-A_3) pre-rRNAs processing, as both precursors accumulate in the mutants (detected by r1/r2 and r5/r6 primers and probes S1–S3). Given its potential role in P′ cleavage in the ITS1-first pathway, Npa1L1 may also cleave the same site in the 5′ETS-first pathway.

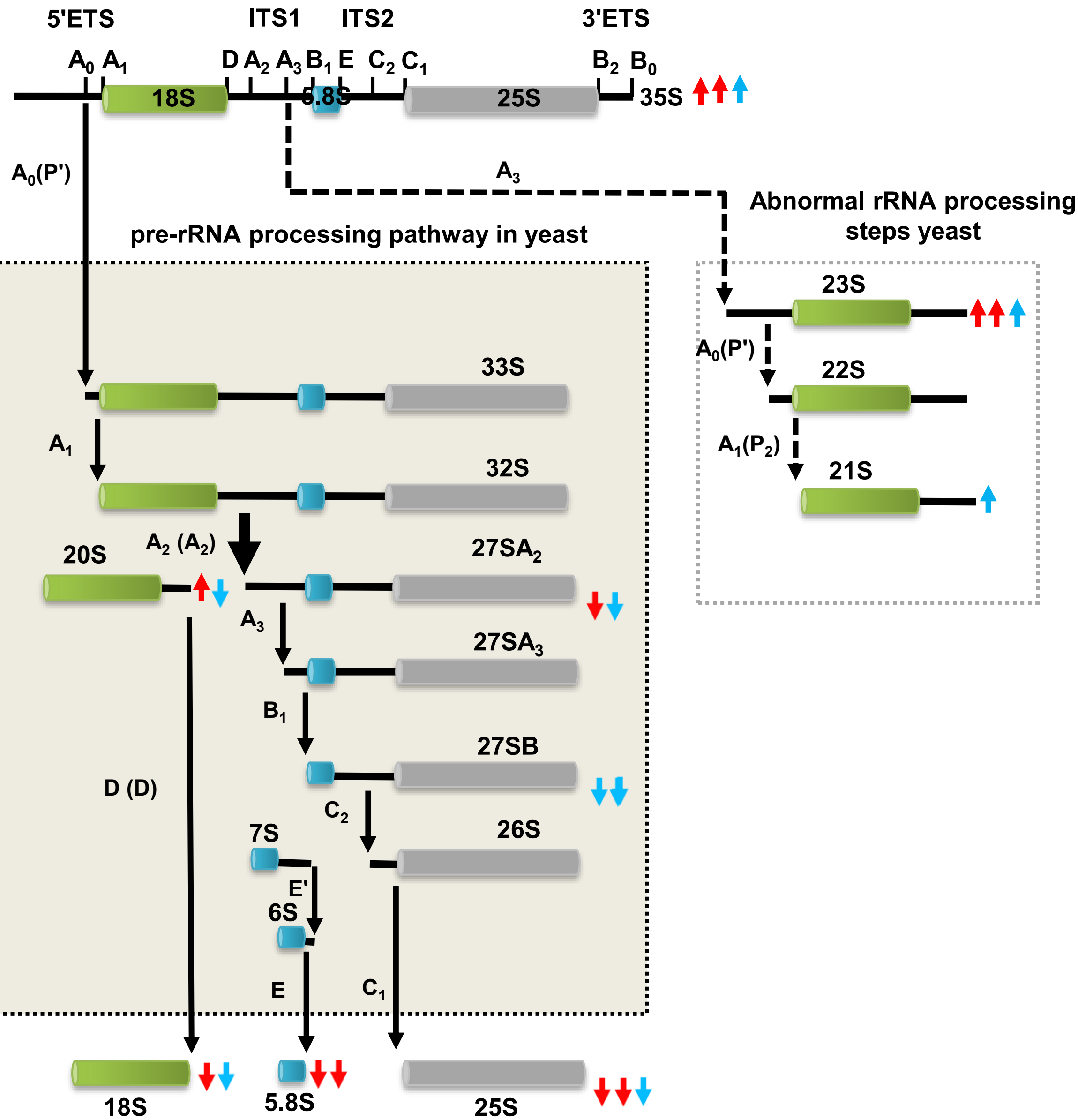

Figure S9. Summary of the roles of yeast Npa1p in pre-rRNA processing (Dez et al., 2004; Rosado and De La Cruz, 2004; Choque et al., 2018; An et al., 2024).

Loss of yeast Npa1p function results in aberrant accumulation or altered processing of several rRNA precursors, including 35S pre-rRNA, $27SA_2$ pre-rRNA, and the aberrant 23S intermediate. In *npa1p* mutants, the levels of $27SA_2$ intermediates are decreased, which is caused by abnormal cleavage at the A_2 site. Meanwhile, the 35S pre-rRNA and 18S rRNA precursors (23S) are overaccumulated and 18S rRNA was reduced, resulting from abnormal cleavage at the A_0, A_3, and D sites, respectively. Red arrows indicate the pre-rRNAs that are overaccumulated or depleted in *npa1p* mutants (Dez et al., 2004). Blue arrows indicate the pre-rRNAs that are overaccumulated or depleted in *npa1p* mutants (Rosado and De La Cruz, 2004). Single red or blue arrow indicates mild alteration in pre-rRNAs abundance, while double arrows denote significant change. The sites in parentheses correspond to the corresponding sites in Arabidopsis.

Table S1. Primers used for identification of T-DNA insertion mutants and amplification of cRT-PCR, and probes sequences for hybridization of northern blot

Primer name	Sequence	Purpose
npa1l1-1-FP	TTCGATTGTTAGACGTGGTCC	For identifying the genotype of *npa1l1-1* mutant plants
npa1l1-1-RP	GCTCAACCGAGCATCTATGAG	
npa1l1-2-FP	AATTTTATCAAAGCCGTTGCTA	For identifying the genotype of *npa1l1-2* mutant plants
npa1l1-2-RP	ATTCCACAGGTTCAATGTAACC	
rh7-3-FP	CAGGGAAAGTGGAGGATTCTACT	For identifying the genotype of *rh7-3* mutant plants
rh7-3-RP	GAATCGTAGAGTGTAACCGCAAC	
rh27-2-FP	TGTAGACCCAATTTCGGTTTG	For identifying the genotype of *rh27-2* mutant plants
rh27-2-RP	TGGGCAAATAACAAGAACACC	
nsn1-1-FP	ACTTTCTTCTGAAGGGCGAAG	For identifying the genotype of *nsn1-1* mutant plants
nsn1-1-RP	TTCGAGGCAGTTGATGATTTC	
Npa1L1-genome-FP	GCAGTAGCAGAACTTATCCAAATCAAG	For identifying genotype of *npa1l1* mutant in complementary assay
Npa1L1-genome-RP	AAATGGTGCAATGACAATGACG	
LBb1.3	ATTTTGCCGATTTCGGAAC	Universal primer for genotyping of T-DNA insertion mutants
18c	ATGCGTCCCTTCCATAAGTC	cRT-PCR primer for reverse transcription of 18S rRNA precursors
25c	CCGTTACTAAGGGAATCCTTGTTAG	cRT-PCR primer for reverse transcription of 25S rRNA precursors
r1	ATGCGTCCCTTCCATAAGTC	For amplifying reverse transcription products with 25c in cRT-PCR
r2	GCAGACGACTTAAATACGCGAC	
r3	GTTCGCAGCACAGCATCC	
r4	CTCCGCTTATTGATATGCTTAAAC	
r5	TGCATGGCTTAATCTTTGAGAC	For amplifying reverse transcription products with 18c in cRT-PCR
r6	AGGAAGGAGAAGTCGTAACAAG	
S1	CGATCACCACTCATACGCCGAACG	Probe for hybridization of northern blot
S2	GGTCGTTCTGTTTTGGACAGGTATC	
S3	GGACTTTGGGTCATCTACAGCTT	

Table S2. Primers used for constructs

Primer name	Sequence	Purpose
pNpa1L1::GFP-Npa1L1-Pro-FP	CTGCAGAACTTCGAGAAATTAGAGAATCGGACCTAA	For amplifying the promoter sequence of *Npa1L1* in complementary assay
pNpa1L1::GFP-Npa1L1-Pro-RP	TCTAGACATCAAAAGAACACTACAGAGAACTCTTCTCTCT	
pNpa1L1::GFP-Npa1L1-genome-FP	GGATCCATGGCGGAATCAATATCATCAGACTTG	For amplifying the genomic DNA of *Npa1L1* in complementary assay
pNpa1L1::GFP-Npa1L1-genome-RP	CCCGGGGGTCTCAACTAAACTTCTTTCCCATTCG	
p35S::GFP-Npa1L1-genome-FP	GGATCCATGGCGGAATCAATATCATCAGACTTG	For amplifying the genomic DNA of *Npa1L1* in co-immunoprecipitation assay
p35S::GFP-Npa1L1-genome-RP	CCCGGGGGTCTCAACTAAACTTCTTTCCCATTCG	
p35S::RH7-MYC-FP	CTGCAGATGCCTTCCCTAATGTTATCTGATA	For amplifying the CDS sequence of *RH7* in co-immunoprecipitation assay
p35S::RH7-MYC-RP	GGATCCATATCTCTGGCCTCTACCACCA	
p35S::RH27-MYC-FP	CTGCAGATGGCGAATTTGGATATGGA	For amplifying the CDS sequence of *RH27* in co-immunoprecipitation assay
p35S::RH27-MYC-RP	TCTAGAGTACCTCTCGAACTTGCTTTTACC	
p35S::NSN1-MYC-FP	CTGCAGATGGTGAAACGGAGTAAAAAGAGTAAG	For amplifying the CDS sequence of *NSN1* in co-immunoprecipitation assay
p35S::NSN1-MYC-RP	TCTAGATTCTTCAGGCAAAAGTCCAGC	
